# Prognostic Factors for Repair Integrity After Arthroscopic Rotator Cuff Repair: A Systematic Review and Meta-analysis

**DOI:** 10.1177/23259671261455856

**Published:** 2026-07-08

**Authors:** Lieselotte Pichler, Mélody Mussard, Michael Oyewale, Laurent Audigé, Christian Appenzeller-Herzog, Andreas Marc Müller, Thomas Stojanov

**Affiliations:** † Department of Orthopaedic Surgery and Traumatology, University Hospital Basel, Basel, Switzerland; ‡ Surgical Outcome Research Center, University Hospital Basel, Basel, Switzerland; § Department of Information and Communications Technologies, Schulthess Klinik, Zurich, Switzerland; ‖ Department of Rehabilitation Medicine, Erasmus MC, University Medical Center Rotterdam, Rotterdam, the Netherlands; ¶ University Medical Library Basel, University of Basel, Basel, Switzerland; Investigation performed at the Department of Orthopaedic Surgery and Traumatology, University Hospital Basel, Basel, Switzerland

**Keywords:** general, muscle injuries, repair integrity, rotator cuff, prognostic factors, shoulder

## Abstract

**Background:**

Repair integrity is the most frequently assessed objective outcome after arthroscopic rotator cuff repair (ARCR). Multiple studies evaluated independent prognostic factors for repair integrity. However, there is a lack of a comprehensive quantitative review summarizing these findings.

**Purpose:**

To provide a descriptive synthesis of the published association estimates between candidate prognostic factors and repair integrity after ARCR.

**Study Design:**

Systematic review; Level of evidence, 4.

**Methods:**

The EMBASE, Medline, and Scopus databases were screened (January 2014-March 2024) for longitudinal studies with multivariate analyses and at least 6 months of follow-up (registration number: CRD42020199257). Association measures were converted to correlation coefficients (*r*) and pooled using a random-effects meta-analysis of prognostic factors and repair integrity, with definitions from individual studies. Data extraction, evaluation of the risk of bias in the included studies, and the certainty of the synthesized evidence were assessed by pairs of study authors using the Checklist for Critical Appraisal and Data Extraction for Systematic Reviews of Prediction Modeling Studies for Prognostic Factors (CHARMS-PF), Quality in Prognosis Study (QUIPS), and Grading of Recommendations, Assessment, Development, and Evaluations (GRADE) tools, respectively.

**Results:**

Of 10,568 records, 30 studies met the inclusion criteria, representing 13,161 patients (mean age, 59 years; female participants: 45%). Recurrent defect rates ranged from 5% to 43%. The meta-analysis included 45% (92/206) of reported association measures, covering 15 factors across 27 studies. There was high-quality evidence supporting that increased age (*r* = 0.02 [95% CI, 0-0.04]) was associated with higher recurrent defect rates. There was moderate quality evidence supporting that larger tear size (*r* = 0.35 [95% CI, 0.21-0.45]), reduced acromiohumeral distance (*r* = 0.32 [95% CI, 0.12-0.50]), greater degree of fatty infiltration of the supraspinatus (*r* = 0.63 [95% CI, 0.14- 0.81]), and greater tear retraction (*r* = 0.30 [95% CI, 0.15-0.42]) were associated with higher rates of recurrent defects. However, there was substantial heterogeneity across studies. The quality of evidence was deemed low to very low for most of the studied factors. Most included studies had a high risk of bias related to confounding, analysis, and reporting.

**Conclusion:**

Moderate- to high-certainty evidence supports 5 prognostic factors—including sociodemographic and diagnostic-related variables—as being associated with higher recurrent defect rates. These results may be used to prioritize further prognostic research studies.

Rotator cuff repair is one of the most frequently performed orthopaedic interventions and is now primarily performed arthroscopically.^74^ Over the past decade, numerous patient-, diagnosis-, and procedure-related risk factors have been reported to influence repair integrity after arthroscopic rotator cuff repair (ARCR).^1^ In the literature, recurrent defect rates vary widely, ranging from 6% to 90%, largely due to inconsistencies in the definition of tendon defects and differences in follow-up duration.^52^ Recurrent defects, when accompanied by worsening clinical and patient-reported outcomes, may require revision surgery,^15^ which can be psychologically distressing for patients and technically demanding for surgeons.^35^

Understanding how prognostic factors, defined as any variable associated with a subsequent outcome, such as repair integrity, is essential before incorporating them into clinical prediction models, which aim to enhance medical decision-making.^60,62^ In the long term, such models can support the identification of patients at high risk of poor outcomes and help prevent complications associated with surgical interventions.^2,63^ Although tear size and fatty infiltration are well-established prognostic factors associated with higher recurrent defect rates after ARCR,^41^ there is a lack of studies providing a comprehensive overview of recent evidence regarding other prognostic factor estimates for repair integrity after ARCR.^49,52^

The present systematic review and meta-analysis aims to provide a descriptive synthesis of published estimates of associations between prognostic factors and repair integrity after ARCR.

## Methods

This review was conducted according to the updated PRISMA (Preferred Reporting Items for Systematic Reviews and Meta-Analyses) guidelines.^54^ The protocol was registered in PROSPERO on August 24, 2020 (registration number: CRD42020199257). This study was partly supported by a Swiss National Science Foundation grant (ID 320030_184959 /1). The funder had no role in study design, data collection, data analysis, data interpretation, or writing of the report.

### Eligibility Criteria

We included prospective and retrospective longitudinal studies of patients undergoing primary ARCR for rotator cuff tears. Studies were eligible if they (1) reported a multivariable model for repair integrity, and (2) reported a clinical follow-up period of at least 6 months. We excluded studies if they (1) involved patients with irreparable tears, (2) involved revision surgeries, or (3) were not published in English, French, or German.

### Information Sources and Search Algorithm

The search strategies were developed by 2 information specialists (including C.A.H.) and peer-reviewed by a third information specialist.^64,65^ Text word synonyms and database-specific subject headings for rotator cuff tear and arthroscopic repair surgery were used to search the electronic databases—Embase (Elsevier), Medline (Ovid), and Scopus (Elsevier). No language restrictions were applied; however, conference abstracts were excluded (Supplementary File 1; last search March 26, 2024).

As surgical rotator cuff repair techniques shifted^13,49^ substantially toward arthroscopy around 2013 and 2014, we restricted the search to studies published after 2014. References were exported to EndNote 21 (Clarivate Analytics) and deduplicated using the Bramer method.^6^

To complement the results of direct database searching, the bibliographic references and citing articles of included articles were retrieved from Lens.org (via citationchaser^27^), Scopus, and Web of Science, then deduplicated as described above and screened for eligibility as described below (March 26, 2024). The bibliographic references of identified systematic and narrative reviews on ARCR were also screened as additional sources. These procedures for backward and forward citation searching were conducted in accordance with the Transparent Reporting of Adaptive Research in Citation Searching (TARCiS) statement.^31^

### Study Selection, Data Collection, and Risk of Bias

Study selection was conducted in 2 phases: title and abstract screening, followed by full-text screening. Both phases were performed independently by at least 2 authors using REDCap^28^ (M.M., L.P., or T.S.), with input from a senior author (T.S.) when disagreements arose.

Data extraction items (see Supplementary File 2) were adapted from the Checklist for Critical Appraisal and Data Extraction for Systematic Reviews of Prediction Modeling Studies for Prognostic Factors (CHARMS-PF).^51^ Specific items related to outcome definition were extracted; notably, the definition or scale used, the type of diagnostic tool employed, and the time point of assessment. Exploratory meta-regression analyses were performed to assess the relative contribution of study-level outcome-definition parameters to the heterogeneity in the observed proportion of recurrent defects.^3^

Data extraction and risk-of-bias assessment were conducted independently by the 2 primary authors (L.P. and M.M.) using the Quality in Prognosis Study (QUIPS) tool.^29^ To inform the items “study population” and “intervention” of the risk of bias assessment, we evaluated the availability of a set of predefined key characteristics describing the study population (tear pattern and tear cause), the intervention (number of surgeons involved and repair technique), and the rehabilitation protocol (duration of postoperative immobilization).

### Summary Measures and Synthesis of Results, Including Meta-analysis

To be included in the quantitative analysis, association measures were required to be (1) adjusted in a multivariable model, including at least 3 factors; and (2) reported with at least 2 of the following items: effect estimate (eg, odds ratio [OR] and regression coefficient), 95% CI, standard deviation, or significance level (eg, *P* = .05). Only prognostic factor association measures that included both a multivariable effect estimate and an accuracy metric (eg, 95% CI or SD) were considered for conversion on a standardized correlation coefficient (*r*).^11^ The transformation from the correlation coefficient to Fisher *Z* (allowing the meta-analysis) was computed using the following formula^21^:

$$\begin{array}{c} z=\frac{1}{2}\ln\left(\frac{1+r}{1-r}\right),\;\mathrm{where}\;r\;\mathrm{refers}\;\mathrm{to}\;\mathrm{the}\;\mathrm{correlation}\;\mathrm{coefficient}. \end{array}$$

Fisher *r*-to-*z* transformations were done using the rma.uni() function from the metafor package in R,^72^ with 'measure' set to “ZCOR” and 'method' to “DL” for the DerSimonian and Laird approach.^14^ Fisher *z* was then back-transformed to correlation coefficients for interpretation. We present the challenges and detailed processes for conducting meta-analyses of prognostic factor estimates in Supplementary File 4. To summarize the strength of association between each prognostic factor and repair integrity, random-effects meta-analysis (using DerSimonian-Laird method^14^) of correlation coefficients was conducted when more than 3 studies reported multivariable associations for the same prognostic factor. The pooled correlation coefficient was the estimate of the meta-analysis. The pooled correlation coefficients were interpreted on a -1 to 1 scale, with higher values indicating greater association with recurrent defects. We used a random-effects model that allowed for the quantification of (1) statistical heterogeneity across studies (using *I*^2^), and (2) pooled association estimates with 95% CIs and prediction intervals.

Forest plots were generated to display the individual and pooled correlation coefficients with 95% CIs, author names, publication years, and study weights. Heterogeneity (*I^2^*) was categorized as low (*I^2^* < 25%), moderate (25%-75%), or high (>75%). Lastly, funnel plots were generated to assess publication bias when ≥10 studies reported an association measure for the same prognostic factor.^59,60^

### Certainty of the Synthesized Evidence

For each reported prognostic factor, and to support interpretation of the findings, the certainty of the synthesized evidence was assessed using an adapted version of the Grading of Recommendations, Assessment, Development, and Evaluations (GRADE) framework for prognostic factor findings.^22,32^ GRADE assessment included the identification of serious limitations for each of the following items: study limitations, inconsistency, indirectness, imprecision, publication bias, and moderate/large effect size. For each prognostic factor, the overall quality of evidence was rated as follows: high if ≥5 items demonstrated no serious limitations; moderate if 3 to 4 items did; low if 1 to 2 items did; and very low if none did.

## Results

After screening of 10,568 titles and abstracts derived from database and citation searching, 732 full-text publications were reviewed for eligibility (Supplementary Figure 1). Notably, 113 full-text articles were excluded for lacking multivariable models, and 70 did not report any estimates of prognostic factors. A total of 30 studies—29 identified through database searching and 1 via citation searching—met the inclusion criteria, encompassing a total of 13,161 patients.^**^

Of the included studies, 93% (n = 28) used retrospectively collected data (Table 1; see also Supplementary Table 1 for detailed extracted items). The analyzed sample sizes ranged from 44 to 1962 patients.^17^ Across studies, the mean age of included patients was 59 years (range, 55 years^23^ to 67 years^61^). The proportion of female participants ranged from 23 (13%) to 47 (77%). Single-row repair was the most commonly used surgical repair technique (n = 14, 47%).**^††^** Most of the studies described data for 1 surgeon (n = 23; 77%).**^‡‡^**

**Table 1:** Synthesized Baseline Study Characteristics and Operative Details*^*a*^*

Synthesized Study Characteristics	N = 30	References
Study sample size, No. of patients		
1-100	8 (27)	^7,18,23,34,44,53,56,69^
101-500	15 (50)	^5,8,10,30,33,38,39,43,45,47,55,57,61,71,73^
501-2000	7 (23)	^16,17,40,42,48,68^
Design		
Prospective	2 (6.7)	^8,55^
Retrospective	28 (93)	^5,7,10,16-18,23,26,30,33,34,38-40,42-45,47,48,53,56,57,61,68,69,71,73^
Tear cause		
All types, degenerative and traumatic	17 (57)	^5,7,8,10,18,23,26,33,39,42,45,53,55,56,68,71,73^
Degenerative	6 (17)	^34,43,44,57,61^
Not described	8 (27)	^16,17,30,38,40,47,48,69^
Involved rotator cuff tendons		
Multiple tendons involved	18 (60)	^8,10,16,18,26,30,33,38-40,42,43,47,48,56,61,71,73^
Supraspinatus only	10 (33)	^5,7,34,44,45,53,55,57,68,69^
Subscapularis only	2 (6.5)	^17,23^
Tear pattern		
Full- and partial-thickness tear	19 (63)	^5,7,8,10,16,18,23,30,33,38,39,42,47,48,56,57,61,71,73^
Full-thickness	8 (27)	^17,34,40,44,45,53,55,69^
Not described	3 (10)	^26,43,68^
No. of surgeons involved		
One surgeon	23 (77)	^5,8,10,16-18,23,26,30,38-40,42-44,47,48,55,56,61,68,69,73^
Several surgeons, 2 to 65	4 (13)	^7,33,57,71^
Not described	3 (10)	^34,45,53^
Repair technique		
Single-row and/or double-row	2 (6.7)	^39,55^
Single-row	14 (47)	^8,16,17,23,26,40,42,43,45,48,56,68,69,71^
Double-row	10 (33)	^5,7,10,18,33,34,38,53,61,73^
Transosseous-equivalent	2 (6.7)	^44,47^
Not described	2 (6.7)	^30,57^
Immobilization period, weeks		
4-6	22 (73)	^5,7,8,10,17,18,26,30,33,38,40,42-44,47,48,53,55,56,61,68,69^
<4	5 (17)	^16,23,39,45,71^
Not described	3 (10)	^34,57,73^

a Data are presented as n (%).

All included studies reported repair integrity as a dichotomized outcome and used multivariable regression analysis to report association measures. Repair integrity was evaluated either using imaging, clinical evaluation, or a combination of both (Table 2). Of the 28 studies that reported the use of image-based evaluation, 19 (68%) defined recurrent defects according to the classification of Sugaya et al^66,67^ for magnetic resonance imaging or computed tomography angiography.^4^ One study^69^ followed a definition by Cho et al^9^ for classifying recurrent defects (see Supplementary Table 1 for complete study outcome definitions).

**Table 2:** Synthesized Postoperative Study Outcomes and Statistical Analyses*^*a*^*

Synthesized Outcome Description	N = 30	References
Outcome evaluation method		
Image-based, MRI/CTA	19 (63)	^7,8,10,18,34,38-40,43,44,47,53,55-57,61,69,71,73^
Image-based, US	8 (27)	^5,16,17,23,26,42,48,68^
Image-based and clinical	1 (3.3)	^45^
Clinical	2 (6.7)	^30,33^
Recurrent defect definition		
Sugaya, types 4-5	18 (60)	^5,7,8,10,18,23,34,38-40,43,47,53,55,56,61,71,73^
Sugaya, types 3-5	1 (3.3)	^44^
Sugaya mentioned	1 (3.3)	^69^
Symptomatic	2 (6.7)	^33,45^
No classification reported	8 (27)	^16,17,26,30,42,48,57,68^
Outcome assessment timepoint, months		
6	11 (37)	^16,17,26,38,42,43,45,47,48,68,71^
12	13 (43)	^5,10,18,33,34,40,44,53,55-57,69,73^
24	6 (20)	^7,8,23,30,39,61^
Variable selection procedure		
Data-driven	16 (53)	^8,18,23,26,34,38,40,43-45,48,55,56,61,71,73^
Expert knowledge	10 (33)	^5,7,16,17,39,42,53,57,68,69^
Not described	4 (13)	^10,30,33,47^

a Data are presented as n (%). CTA, computed tomography angiography; MRI, magnetic resonance imaging; US, ultrasound.

Across studies, the reported recurrent defect rate was 14% (n = 1923/13,214), ranging from 5% to 43%. Most of the heterogeneity in reported recurrent defect rates was explained by outcome definition parameters (evaluation time point and evaluation outcome methods listed in Table 2, *R*^2^ = 53%), with lower recurrent defects rates observed when study authors used an ultrasound image-based evaluation (β = −0.45 [95% CI, −0.91 to −0.008]; *P* < .0001) or a symptomatic evaluation (β =–1.26 [95% CI, −1.93 to −0.579]; *P* = .0003). Demographic, diagnostic, study- and treatment-related factors explained between 0% and 12% of the observed heterogeneity (Table 1).

A total of 206 individual associations between 60 unique prognostic factors and repair integrity were reported. Further details on the types, definitions, and handling of these prognostic factors, as extracted from the original articles, are available in Supplementary Table 2. Only 40% (n = 24) of the individual prognostic factors were reported at least 3 times across studies. Notably, 72 of the 206 reported associations (35%) did not include any association measure due to insufficient reporting.

**Figure 1.**
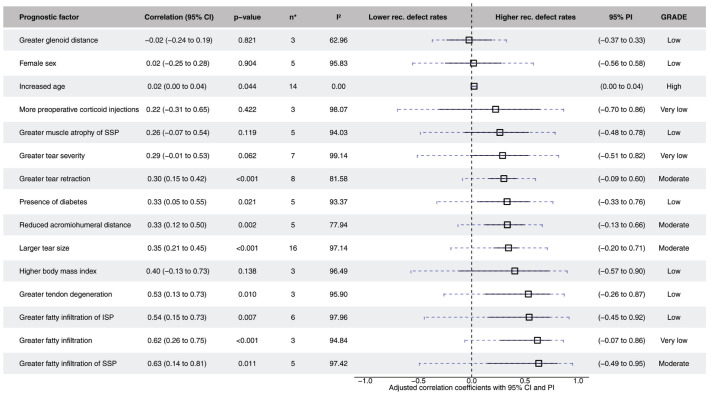
Meta-analysis of multivariable association estimates. Prognostic factors for which at least 3 association measures were reported were considered for meta-analysis. Full definitions of reported prognostic factors are provided in Supplementary Table 2. Meta-analyzed correlation coefficients are presented with their 95% CIs (black lines) and 95% prediction intervals (dashed blue lines). The *P* value is the result of a statistical test assessing the difference between 0 and the correlation coefficient. n* refers to the number of reported association measures across studies. *I*^2^ indicates heterogeneity (in %) from the individual random-effect meta-analysis model. A full description of the items for the GRADE assessment for each prognostic factor is provided in Supplementary Table 4. GRADE, Grading of Recommendations, Assessment, Development, and Evaluations; ISP, infraspinatus; PI, prediction interval; Rec, recurrent; SSP, supraspinatus.

In summary, 45% (92/206) of the reported association measures about 15 prognostic factors across 27 studies**^§§^** were included in the meta-analysis (Figure 1, Supplementary Figures 2). Heterogeneity was very low for age (*I^2^* = 0%) (Figure 1). For all other factors, heterogeneity was moderate to high (*I^2^* = 62% for glenoid distance to 99% for tear severity).

**Figure 2.**
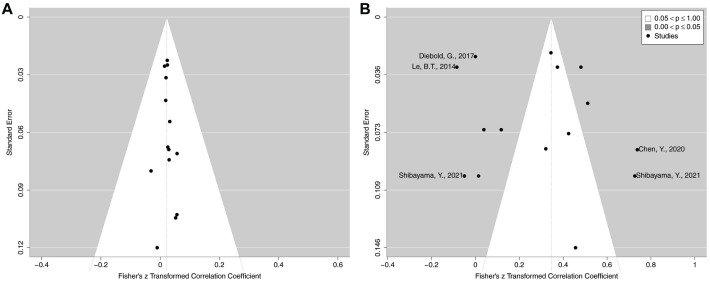
Publication bias was assessed using funnel plots. (A) Age. (B) Tear size. Funnel plots were generated to assess potential publication bias when >10 association measures for the same prognostic factor and repair integrity were reported.

There was high- and moderate-quality evidence supporting that increased age, greater tear retraction, reduced acromiohumeral distance, larger tear size, and greater degree of fatty infiltration of the supraspinatus were associated with higher recurrent defect rates (Figure 2 and Supplementary Table 4). The quality of evidence was rated low for body mass index, diabetes, fatty infiltration of the rotator cuff (without differentiation of the affected tendon), fatty infiltration of the infraspinatus, female sex, glenoid distance, and muscle atrophy of the supraspinatus. The quality of evidence was very low for preoperative corticoid injections and tear severity. While funnel plot inspection was possible for age and tear size, only the latter suggested asymmetry (Figure 2B). Results of the Egger test were not significant for age (*t* = 0.5639, p = 0.5832) or tear size (*t* = 1.0660; *P* = .3044). The quality of evidence was rated low and very low for 7 and 2 prognostic factors, respectively.

Of note, while the meta-analysis of correlation coefficients might have revealed significant pooled correlation coefficients associated with repair integrity, the prediction intervals still overlapped with zero, indicating substantial heterogeneity across studies (as supported by the high I2 values, except for age). This suggests that, despite statistically significant pooled estimates, the true effect of the prognostic factors may vary considerably in future similar research.

**Figure 3.**
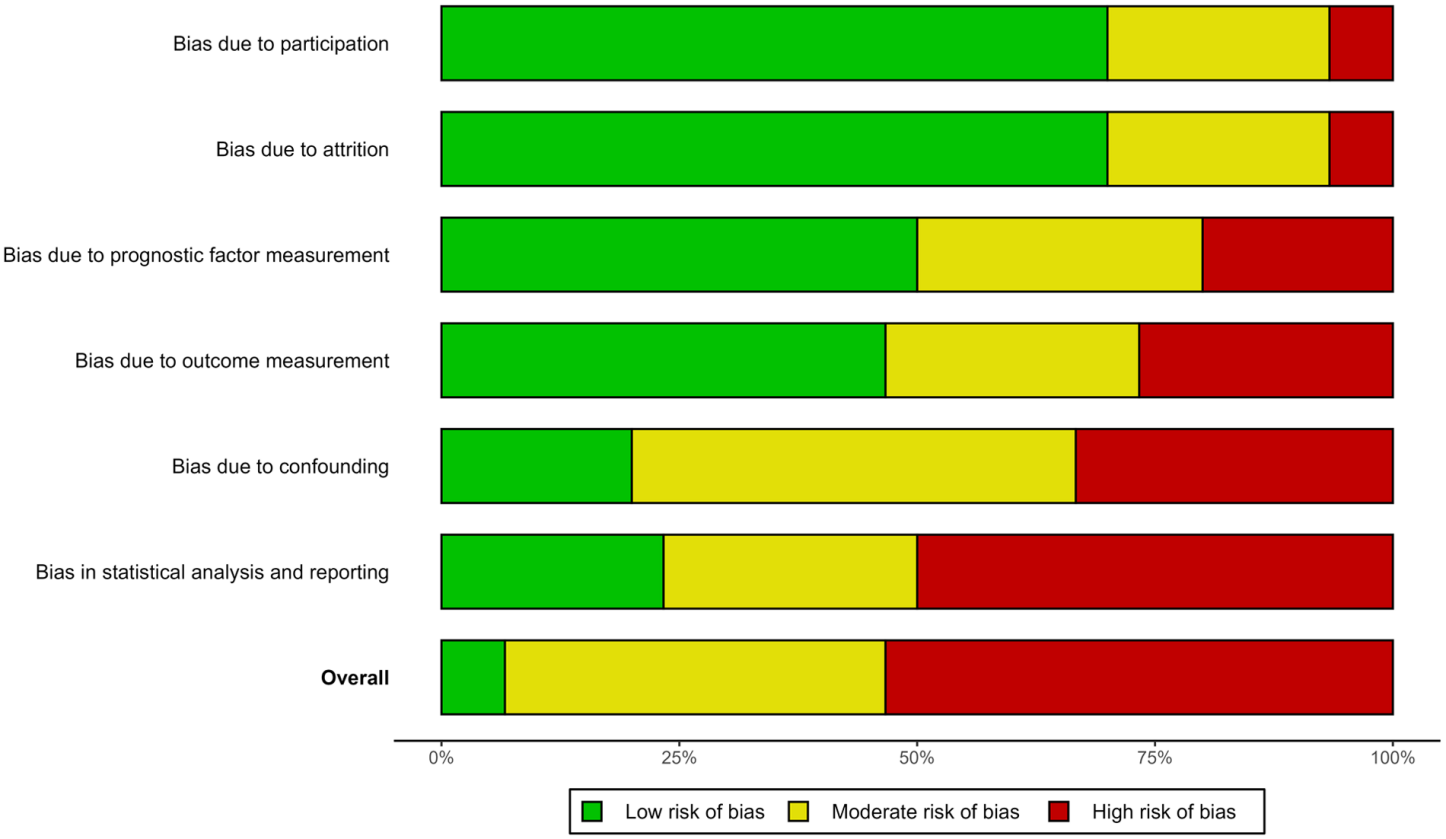
Risk of bias in included studies.

Most of the included studies demonstrated a low risk of bias with respect to study population (n = 20; 67%) and attrition (n = 21; 70%) (Figure 3 and Supplementary Table 3). In contrast, a moderate or high risk of bias was identified in half of the included studies for the measurement of the prognostic factor (n = 15; 50%) and the outcome (n = 16; 53%). Notably, the classification of outcomes was not clearly described in 27% of studies (n = 8). The proportion of included studies with a moderate or high risk of bias, due to (1) confounding and (2) statistical analysis and reporting, was 80% and 76%, respectively—mostly due to poor reporting (see Supplementary Table 2).

## Discussion

This systematic review and meta-analysis synthesized evidence from 30 studies that described associations between prognostic factors and repair integrity after ARCR. Reported recurrent defect rates ranged from 5% to 43% and were impacted by the method of evaluation, with ultrasound or symptomatic evaluation highlighting lower rates.^52^

The quantitative analysis, which included 27 studies, revealed methodological limitations and should be interpreted with caution. Our findings highlighted high-quality evidence indicating that older age is associated with higher rates of recurrent defects after ARCR. Moderate-quality evidence supported associations between larger preoperative tear size, reduced acromiohumeral distance, greater tear retraction, a greater degree of fatty infiltration of the supraspinatus, and higher recurrent defect rates after ARCR. Notably, greater global and infraspinatus fatty infiltration, more extensive tendon degeneration, and diabetes were also associated with higher rates of recurrent defects. However, the quality of evidence for these associations was low. Similarly, low-quality evidence suggests that body mass index, sex, and glenoid distance are not associated with repair integrity. The low-quality evidence for the individual variables limits the ability to draw clear conclusions about their prognostic relevance. These findings highlight the need for further high-quality prognostic research using robust modeling and reporting standards. We provide the clinical contexts below that support the interpretation of the synthesized association measures, which are not intended as causal explanations.

Although the pooled correlation coefficient for increased age was relatively small, the meta-analysis identified a statistically significant association between older age and higher rates of recurrent defects after ARCR. All included studies reported ORs for age (treated as a continuous variable, with 1-year increments) and repair integrity. Clinically, age is usually associated with degenerative tendon alterations, larger tear size, and a higher degree of fatty infiltration.^46^ Increased age is associated with poorer recovery or functional outcomes after ARCR in previous systematic reviews.^20,41,64^ Of note, all included studies reported ORs for age (treated as a continuous factor using a 1-year increase unit). The vast majority of studies also reported continuous variables for tear size (using a 1-mm or 1-cm increase) and muscle atrophy (using a 1% increase). Interpretation of such factor-handling and related association measures assesses whether an increase in the unit of the variable (here, years, cm/mm, or % of atrophy) is associated with better or worse outcomes. One should note that the aim of the meta-analysis was not to identify cutoffs (eg, age >50 or <0 years) that maximize prediction performance in each study, but rather to highlight trends in the consistency of reported association measures across studies.

Other studies have demonstrated that full-thickness tear size correlates with increased repair tension.^37,49^ Minimizing rotator cuff tension is a well-established surgical goal in ARCR, and high repair tension has been shown to correlate with poorer clinical outcomes after ARCR. However, only one study in this review provided a direct measure of the association between muscle tension and repair integrity, reporting a large effect size.^56^ The observed association between larger tear size and higher rates of recurrent defects highlighted in the present review is consistent with previous literature reviews in the field.^20,41^ Further multivariable analyses are therefore warranted to confirm the strength of this association, particularly in the context of muscle tension indicators.

Half of the studies included in the present review reported association measures describing tendon degeneration or fatty infiltration.**^‖ ‖^** Tear retraction, a higher degree of fatty infiltration, and tendon degeneration more generally, are established prognostic factors associated with an increased occurrence of recurrent defects after ARCR. The findings of this systematic review confirmed that greater tear retraction, which may increase with time to surgery, was associated with higher rates of recurrent defects. On the one hand, tear retraction is a good indicator of reduced muscle quality and elasticity.^70^ On the other hand, fatty accumulation is associated with markers of poorer metabolic health and degeneration of the tendon.^25^

In this review, we found moderate-certainty evidence supporting an association between a reduced acromiohumeral distance and increased rates of recurrent defects after ARCR. Clinically and biomechanically, a reduced acromiohumeral distance often reflects a major rotator cuff tear, as, without the lowering and centering effect of the rotator cuff on the humeral head, the humeral head migrates superiorly.^36,58^ There is also a strong association between reduced acromiohumeral distance and fatty infiltration of the infraspinatus tendon.^24^

Patients with uncontrolled diabetes may exhibit higher recurrent defect rates, as altered metabolic processes may also play a significant role in rotator cuff healing.^9^ The results of our meta-analysis have supported this statement. However, the low quality of the synthesized evidence limits the strength of conclusions regarding the prognostic relevance of diabetes.

Glenoid distance, number of preoperative corticoid injections, tear severity, and muscle atrophy of the supraspinatus were not significantly associated with repair integrity. These prognostic factors were inconsistently defined across studies, leaving room for improvement in the standardization of terminology. Sex was also consistently not associated with repair integrity, although there is growing interest in sex-specific outcome data after ARCR.^50^ This finding aligns with the conclusions of a recent systematic review.^19^ However, caution is warranted when interpreting this result as the proportion of female participants was widely varied across studies, ranging from 13% to 77%. An underestimation of the association between sex and outcomes after ARCR cannot currently be excluded.^50^

Similarly, in the present review, 4 studies reported associations between surgical techniques and repair integrity.^7,33,39^ However, the reported measures of association were inconsistently reported and encompassed a range of concepts, including suture technique configurations^33,39,53^ and additional procedures, such as tenodesis or lateral clavicula resection. Because of this heterogeneity and incomplete reporting, surgical technique could not be included in the quantitative analysis, and the strength of its association with repair integrity remains unclear.

### Limitations

Acknowledging the inherent variability in association measures across primary studies, we converted reported association measures—including odds ratios for both continuous and categorical factors— into standardized correlation coefficients. This process involved necessary approximations and contributed to the heterogeneity observed, which was accounted for when grading the certainty of the synthesized evidence and interpreting the results. Because too few studies reported adjusted association measures with the same set of adjustment covariates, this additional filtering step could not be included in the meta-analysis and is therefore acknowledged as a main limitation, limiting the interpretability of the findings, where an adequate covariate adjustment structure (including the assessment of the causal roles of adjusted variables, effect modification, and nonlinearity) would be detrimental.

No sensitivity analyses were performed to assess the robustness of the meta-analysis. Moreover, the interpretation of the findings of this systematic review was limited by (1) the overall risk of bias of the included studies, (2) the substantial heterogeneity observed in several pooled results and outcome definitions, and (3) inadequate reporting of association measures. As a result, many prognostic factors could not be included in the meta-analysis because they were reported in <3 studies. From a predictive research perspective, we did not report performance metrics for the models described, such as discrimination or calibration.

Therefore, we strongly encourage authors of future studies to adhere to established reporting guidelines to enhance transparency, comparability, and inclusion in future meta-analyses.^12^

## Conclusion

The findings of this review highlight that key prognostic factors associated with higher recurrent defect rates after ARCR include both sociodemographic (increased age) and diagnostic-related variables (such as greater tear retraction, larger tear size, reduced acromiohumeral distance, and greater degree of fatty infiltration of the supraspinatus). The described methodology may also be used in future similar studies aimed at summarizing evidence on measures of association with prognostic factors. Our results may be used to prioritize further causal inference or prognostic research studies in the field. For elderly patients or highly degenerative rotator cuff tears, surgical decisions should be discussed thoroughly and transparently before proceeding with ARCR. Given the moderate-to-low quality of the existing evidence, the prognostic value of the identified factors remains to be validated in the development of comprehensive prediction models.

## Supplemental Material

sj-pdf-1-ojs-10.1177_23259671261455856 – Supplemental material for Prognostic Factors for Repair Integrity After Arthroscopic Rotator Cuff Repair: A Systematic Review and Meta-analysis Supplemental material, sj-pdf-1-ojs-10.1177_23259671261455856 for Prognostic Factors for Repair Integrity After Arthroscopic Rotator Cuff Repair: A Systematic Review and Meta-analysis by Lieselotte Pichler, Mélody Mussard, Michael Oyewale, Laurent Audigé, Christian Appenzeller-Herzog, Andreas Marc Müller and Thomas Stojanov in Orthopaedic Journal of Sports Medicine

sj-xlsx-1-ojs-10.1177_23259671261455856 – Supplemental material for Prognostic Factors for Repair Integrity After Arthroscopic Rotator Cuff Repair: A Systematic Review and Meta-analysis Supplemental material, sj-xlsx-1-ojs-10.1177_23259671261455856 for Prognostic Factors for Repair Integrity After Arthroscopic Rotator Cuff Repair: A Systematic Review and Meta-analysis by Lieselotte Pichler, Mélody Mussard, Michael Oyewale, Laurent Audigé, Christian Appenzeller-Herzog, Andreas Marc Müller and Thomas Stojanov in Orthopaedic Journal of Sports Medicine

## Notes

** References 5, 7, 8, 10, 16-18, 23, 26, 30, 33, 34, 38-40, 42-45, 47, 48, 53, 55-57, 61, 68, 69, 71, 73.

†† References 8, 16, 17, 23, 26, 40, 42, 43, 45, 48, 56, 68, 69, 71.

‡‡ References 5, 8, 10, 16-18, 23, 26, 30, 38-40, 42-44, 47, 48, 55, 56, 61, 68, 69, 73.

§§ References 5, 7, 8, 10, 16-18, 26, 30, 33, 34, 38-40, 42-45, 47, 53, 55-57, 61, 69, 71, 73.

‖ ‖ References 5, 8, 10, 33, 39, 40, 43, 44, 45, 53, 55, 56, 61, 71, 73.

## References

[1] Abtahi AM Granger EK Tashjian RZ. Factors affecting healing after arthroscopic rotator cuff repair. World J Orthop. 2015;6(2):211-220. 25793161 10.5312/wjo.v6.i2.211 PMC4363803

[2] Audigé L Aghlmandi S Grobet C , et al. Prediction of shoulder stiffness after arthroscopic rotator cuff repair. Am J Sports Med. 2021;49(11):3030-3039. 34310220 10.1177/03635465211028980

[3] Baker WL Michael White C Cappelleri JC , et al. Understanding heterogeneity in meta-analysis: the role of meta-regression. Int J Clin Pract. 2009;63(10):1426-1434. 19769699 10.1111/j.1742-1241.2009.02168.x

[4] Barth J Fotiadis E Barthelemy R Genna S Saffarini M. Ultrasonic evaluation of the repair integrity can predict functional outcomes after arthroscopic double-row rotator cuff repair. Knee Surg Sports Traumatol Arthrosc. 2015;23(2):376-385. 25600261 10.1007/s00167-015-3505-z

[5] Baverel L Boutsiadis A Reynolds RJ , et al. Do corticosteroid injections compromise rotator cuff tendon healing after arthroscopic repair? JSES Open Access. 2018;2(1):54-59. 30675568 10.1016/j.jses.2017.11.005 PMC6334978

[6] Bramer WM Giustini D de Jonge GB Holland L Bekhuis T. De-duplication of database search results for systematic reviews in EndNote. J Med Libr Assoc. 2016;104(3):240. 27366130 10.3163/1536-5050.104.3.014 PMC4915647

[7] Caffard T Kralewski D Ludwig M , et al. High acromial slope and low acromiohumeral distance increase the risk of retear of the supraspinatus tendon after repair. Clin Orthop Relat Res. 2023;481(6):1158-1170. 36623210 10.1097/CORR.0000000000002520 PMC10194550

[8] Chen Y Jiang F Li H , et al. Retears and concomitant functional impairments after rotator cuff repair: shoulder activity as a risk factor. Am J Sports Med. 2020;48(4):931-938. 32040348 10.1177/0363546519900897

[9] Cho NS Moon SC Jeon JW Rhee YG. The influence of diabetes mellitus on clinical and structural outcomes after arthroscopic rotator cuff repair. Am J Sports Med. 2015;43(4):991-997. 25622985 10.1177/0363546514565097

[10] Choi S Kim MK Kim GM , et al. Factors associated with clinical and structural outcomes after arthroscopic rotator cuff repair with a suture bridge technique in medium, large, and massive tears. J Shoulder Elbow Surg. 2014;23(11):1675-1681. 24862247 10.1016/j.jse.2014.02.021

[11] Cohen J Cohen P West SG Aiken LS. Applied Multiple Regression/Correlation Analysis for the Behavioral Sciences. Routledge; 2013.

[12] Collins GS Moons KGM Dhiman P , et al. TRIPOD+AI statement: updated guidance for reporting clinical prediction models that use regression or machine learning methods. BMJ. 2024;385:e078378. 10.1136/bmj-2023-078378 PMC11019967 38626948

[13] Denard PJ Burkhart SS. The evolution of suture anchors in arthroscopic rotator cuff repair. Arthroscopy. 2013;29(9):1589-1595. 23876609 10.1016/j.arthro.2013.05.011

[14] DerSimonian R Laird N. Meta-analysis in clinical trials. Controlled Clin Trials. 1986;7(3):177-188. 3802833 10.1016/0197-2456(86)90046-2

[15] Desmoineaux P. Failed rotator cuff repair. Orthop Traumatol Surg Res. 2019;105(suppl 1):S63-S73. 10.1016/j.otsr.2018.06.012 30130661

[16] Diebold G Lam P Walton J Murrell GAC . Relationship between age and rotator cuff retear: a study of 1,600 consecutive rotator cuff repairs. J Bone Joint Surg Am. 2017;99(14):1198-1205. 28719559 10.2106/JBJS.16.00770

[17] Duong JKH Lam PH Murrell GAC . Anteroposterior tear size, age, hospital, and case number are important predictors of repair integrity: an analysis of 1962 consecutive arthroscopic single-row rotator cuff repairs. J Shoulder Elbow Surg. 2021;30(8):1907-1914. 33160028 10.1016/j.jse.2020.09.038

[18] Erşen A Şahin K Albayrak MO. Older age and higher body mass index are independent risk factors for tendon healing in small- to medium-sized rotator cuff tears. Knee Surg Sports Traumatol Arthrosc. 2023;31(2):681-690. 36399192 10.1007/s00167-022-07234-6

[19] Fancher AJ Mok AC Vopat ML , et al. Comparing sex-specific outcomes after rotator cuff repair: a meta-analysis. Orthop J Sports Med. 2022;10(5):23259671221086259. 10.1177/23259671221086259 PMC9128058 35620113

[20] Fermont AJ Wolterbeek N Wessel RN Baeyens JP de Bie RA. Prognostic factors for successful recovery after arthroscopic rotator cuff repair: a systematic literature review. J Orthop Sports Phys Ther. 2014;44(3):153-163. 24450368 10.2519/jospt.2014.4832

[21] Fisher RA. On the "probable error" of a coefficient of correlation deduced from a small sample. Metron. 1921;1:3-32.

[22] Foroutan F Guyatt G Zuk V , et al. GRADE guidelines 28: use of GRADE for the assessment of evidence about prognostic factors: rating certainty in identification of groups of patients with different absolute risks. J Clin Epidemiol. 2020;121:62-70. 31982539 10.1016/j.jclinepi.2019.12.023

[23] Galasso O Mercurio M Gasparini G , et al. Arthroscopic rotator cuff repair in patients over 65 years of age: successful functional outcomes and a high tendon integrity rate can be obtained after surgery. JSES Int. 2024;8(2):299-303. 38464433 10.1016/j.jseint.2023.11.010 PMC10920122

[24] Goutallier D Le Guilloux P Postel JM , et al. Acromio humeral distance less than six millimeter: Its meaning in full-thickness rotator cuff tear. Orthop Traumatol Surg Res. 2011;97(3):246-251. 21459063 10.1016/j.otsr.2011.01.010

[25] Goutallier D Postel J-M Gleyze P Leguilloux P Van Driessche S. Influence of cuff muscle fatty degeneration on anatomic and functional outcomes after simple suture of full-thickness tears. J Shoulder Elbow Surg. 2003;12(6):550-554. 14671517 10.1016/s1058-2746(03)00211-8

[26] Guo AA Stitz DJ Lam P Murrell GAC . Tear size and stiffness are important predictors of retear: an assessment of factors associated with repair integrity at 6 months in 1,526 rotator cuff repairs. JBJS Open Access. 2022;7(3). 10.2106/JBJS.OA.22.00006 PMC9509124 36168327

[27] Haddaway NR Grainger MJ Gray CT. Citationchaser: a tool for transparent and efficient forward and backward citation chasing in systematic searching. Res Synth Methods. 2022;13(4):533-545. 35472127 10.1002/jrsm.1563

[28] Harris PA Taylor R Minor BL , et al. The REDCap consortium: Building an international community of software platform partners. J Biomed Inform. 2019;95:103208. 31078660 10.1016/j.jbi.2019.103208 PMC7254481

[29] Hayden JA Côté P Bombardier C. Evaluation of the quality of prognosis studies in systematic reviews. Ann Intern Med. 2006;144(6):427-437. 16549855 10.7326/0003-4819-144-6-200603210-00010

[30] Herring MJ White M Braman JP. The WORC Index and predicting treatment failure in patients undergoing primary arthroscopic rotator cuff repair. Orthop J Sports Med. 2019;7(7):2325967119859518. 10.1177/2325967119859518 PMC6664644 31384619

[31] Hirt J Nordhausen T Fuerst T Ewald H Appenzeller-Herzog C. Guidance on terminology, application, and reporting of citation searching: the TARCiS statement. BMJ. 2024;385. 10.1136/bmj-2023-078384 38724089

[32] Huguet A Hayden JA Stinson J , et al. Judging the quality of evidence in reviews of prognostic factor research: adapting the GRADE framework. Syst Rev. 2013;2:1-12. 24007720 10.1186/2046-4053-2-71 PMC3930077

[33] Johnson AH West M Fowler MB , et al. What is the optimal construct to reduce failure in arthroscopic four anchor rotator cuff repair? Shoulder Elbow. 2023;15(suppl 4):S33-S39. 10.1177/17585732221076066 PMC10649482 37974601

[34] Kang Y Lee GY Lee JW , et al. Texture analysis of torn rotator cuff on preoperative magnetic resonance arthrography as a predictor of postoperative tendon status. Korean J Radiol. 2017;18(4):691-698. 28670164 10.3348/kjr.2017.18.4.691 PMC5447645

[35] Keener JD. Revision rotator cuff repair. Clin Sports Med. 2012;31(4):713-725. 23040555 10.1016/j.csm.2012.07.007

[36] Kholinne E Kwak JM Sun Y , et al. The relationship between rotator cuff integrity and acromiohumeral distance following open and arthroscopic rotator cuff repair. SICOT J. 2021;7:23. 33812470 10.1051/sicotj/2021012 PMC8019552

[37] Kim DH Jang YH Choi YE Lee H-R Kim SH. Evaluation of repair tension in arthroscopic rotator cuff repair: does it really matter to the integrity of the rotator cuff? Am J Sports Med. 2016;44(11):2807-2812. 27400717 10.1177/0363546516651831

[38] Kim MS Rhee SM Cho NS. Increased HbA1c levels in diabetics during the postoperative 3-6 months after rotator cuff repair correlated with increased retear rates. Arthroscopy. 2023;39(2):176-182. 36049586 10.1016/j.arthro.2022.08.021

[39] Kim Y-K Jung K-H Kim J-W Kim U-S Hwang D-H. Factors affecting rotator cuff integrity after arthroscopic repair for medium-sized or larger cuff tears: a retrospective cohort study. J Shoulder Elbow Surg. 2018;27(6):1012-1020. 29290609 10.1016/j.jse.2017.11.016

[40] Kwon J Lee YH Kim SH , et al. Delamination does not affect outcomes after arthroscopic rotator cuff repair as compared with nondelaminated rotator cuff tears: a study of 1043 consecutive cases. Am J Sports Med. 2019;47(3):674-681. 30629459 10.1177/0363546518817764

[41] Lambers Heerspink FO Dorrestijn O van Raay JJ Diercks RL. Specific patient-related prognostic factors for rotator cuff repair: a systematic review. J Shoulder Elbow Surg. 2014;23(7):1073-1080. 24725900 10.1016/j.jse.2014.01.001

[42] Le BTN Wu XL Lam PH Murrell GAC . Factors predicting rotator cuff retears: an analysis of 1000 consecutive rotator cuff repairs. Am J Sports Med. 2014;42(5):1134-1142. 24748610 10.1177/0363546514525336

[43] Lim TK Bae KH Choi YS Kim JH Yoo JC. Clinical outcome and repair integrity after arthroscopic rotator cuff repair significantly improved during the surgeon's learning curve. J Shoulder Elbow Surg. 2021;30(8):1881-1890. 33271322 10.1016/j.jse.2020.10.031

[44] Liu XN Yang C-J Lee GW , et al. Functional and radiographic outcomes after arthroscopic transosseous suture repair of medium sized rotator cuff tears. Arthroscopy. 2018;34(1):50-57. 29079262 10.1016/j.arthro.2017.07.035

[45] Lobo-Escolar L Ramazzini-Castro R Codina-Grañó D , et al. Risk factors for symptomatic retears after arthroscopic repair of full-thickness rotator cuff tears. J Shoulder Elbow Surg. 2021;30(1):27-33. 32862994 10.1016/j.jse.2020.05.010

[46] Long Z Nakagawa K Wang Z , et al. Age-related cellular and microstructural changes in the rotator cuff enthesis. J Orthop Res. 2022;40(8):1883-1895. 34783060 10.1002/jor.25211 PMC9107523

[47] Manop P Apivatgaroon A Puntu W Chernchujit B. Risk factors for rotator cuff repair failure and reliability of the rotator cuff healing index (RoHI) in Thai patients: comparison of the RoHI with a modified scoring system. Orthop J Sports Med. 2023;11(6). 10.1177/23259671231179449 PMC10334006 37441508

[48] McColl AH Lam PH Murrell GAC . Are we getting any better? A study on repair integrity in 1600 consecutive arthroscopic rotator cuff repairs. JSES Open Access. 2019;3(1):12-20. 30976730 10.1016/j.jses.2019.01.002 PMC6443836

[49] McElvany MD McGoldrick E Gee AO Neradilek MB Matsen FA. Rotator cuff repair: published evidence on factors associated with repair integrity and clinical outcome. Am J Sports Med. 2015;43(2):491-500. 24753240 10.1177/0363546514529644

[50] Monteleone AS Salerno M Mondini Trissino da Lodi C , et al. The influence of sex is a neglected focus in rotator cuff repair: a systematic review and meta-analysis. Knee Surg Sports Traumatol Arthrosc. 2024;32(10):2699-2710. 38678392 10.1002/ksa.12201

[51] Moons KG de Groot JA Bouwmeester W , et al. Critical appraisal and data extraction for systematic reviews of prediction modelling studies: the CHARMS checklist. PLoS Med. 2014;11(10):e1001744. 10.1371/journal.pmed.1001744 PMC4196729 25314315

[52] Müller AM Flury M Alsayed HN Audigé L. Influence of patient and diagnostic parameters on reported retear rates after arthroscopic rotator cuff repair. Knee Surg Sports Traumatol Arthrosc. 2017;25(7):2089-2099. 28255656 10.1007/s00167-017-4481-2

[53] Olthof MGL Flück M Borbas P , et al. Structural musculotendinous parameters that predict failed tendon healing after rotator cuff repair. Orthop J Sports Med. 2023;11(9). 10.1177/23259671231196875 PMC10510361 37736603

[54] Page MJ McKenzie JE Bossuyt PM , et al. The PRISMA 2020 statement: an updated guideline for reporting systematic reviews. J Clin Epidemiol. 2021;134:178-189. 33789819 10.1016/j.jclinepi.2021.03.001

[55] Park JS Park HJ Kim SH Oh JH. Prognostic factors affecting rotator cuff healing after arthroscopic repair in small to medium-sized tears. Am J Sports Med. 2015;43(10):2386-2392. 26286879 10.1177/0363546515594449

[56] Park S-G Shim B-J Seok H-G. How much will high tension adversely affect rotator cuff repair integrity? Arthroscopy. 2019;35(11):2992-3000. 31629587 10.1016/j.arthro.2019.05.049

[57] Rashid MS Cooper C Cook J , et al. Increasing age and tear size reduce rotator cuff repair healing rate at 1 year. Acta Orthop. 2017;88(6):606-611. 28880113 10.1080/17453674.2017.1370844 PMC5694804

[58] Razmjou H Veronica P Monique C Susan R and Kennedy D. Reduced acromiohumeral distance and increased critical shoulder angle: implications for primary care clinicians. Phys Sportsmed. 2020;48(3):312-319. 31829074 10.1080/00913847.2019.1703475

[59] Riley RD Moons KG Snell KI , et al. A guide to systematic review and meta-analysis of prognostic factor studies. BMJ. 2019;364. 10.1136/bmj.k4597 30700442

[60] Riley RD van der Windt D Croft P Moons KG. Prognosis Research in Healthcare: Concepts, Methods, and Impact. Oxford University Press; 2019.

[61] Shibayama Y Hirose T Sugi A , et al. Relationship between preoperative size of rotator cuff tears measured using radial-slice magnetic resonance images and postoperative rotator cuff integrity: a prospective case-control study. JSES Int. 2021;6(2):279-286. 35252927 10.1016/j.jseint.2021.11.005 PMC8888162

[62] Steyerberg EW. Clinical Prediction Models. 2009.

[63] Stojanov T Aghlmandi S Müller AM , et al. Development and internal validation of a model predicting patient-reported shoulder function after arthroscopic rotator cuff repair in a Swiss setting. Diagn Progn Res. 2023;7(1):21. 37932868 10.1186/s41512-023-00156-y PMC10629040

[64] Stojanov T Audigé L Modler L , et al. Prognostic factors for improvement of shoulder function after arthroscopic rotator cuff repair: a systematic review. JSES Int. 2023;7(1):50-57. 36820428 10.1016/j.jseint.2022.09.003 PMC9937854

[65] Stojanov T Modler L Müller AM , et al. Prognostic factors for the occurrence of post-operative shoulder stiffness after arthroscopic rotator cuff repair: a systematic review. BMC Musculoskelet Disord. 2022;23(1):1-10. 35090426 10.1186/s12891-022-05030-4 PMC8800355

[66] Sugaya H Maeda K Matsuki K Moriishi J. Functional and structural outcome after arthroscopic full-thickness rotator cuff repair: single-row versus dual-row fixation. Arthroscopy. 2005;21(11):1307-1316. 16325080 10.1016/j.arthro.2005.08.011

[67] Sugaya H Maeda K Matsuki K Moriishi J. Repair integrity and functional outcome after arthroscopic double-row rotator cuff repair: a prospective outcome study. J Bone Joint Surg Am. 2007;89(5):953-960. 17473131 10.2106/JBJS.F.00512

[68] Tan M Lam PH Le B Murrell GAC . Trauma versus no trauma: Effect of tear mechanism in the outcomes of arthroscopic rotator cuff repair in 1300 consecutive patients. J Sci Med Sport. 2014;18(NA):e161-NA. 10.1016/j.jse.2015.06.023 26264504

[69] Tashjian RZ Granger EK Zhang Y Teerlink CC Cannon-Albright LA. Identification of a genetic variant associated with rotator cuff repair healing. J Shoulder Elbow Surg. 2016;25(6):865-872. 27066960 10.1016/j.jse.2016.02.019

[70] Tashjian RZ Hung M Burks RT Greis PE. Influence of preoperative musculotendinous junction position on rotator cuff healing using single-row technique. Arthroscopy. 2013;29(11):1748-1754. 24209672 10.1016/j.arthro.2013.08.014

[71] Tokunaga T Karasugi T Tanimura S Miyamoto T. Association of severe histological degeneration of the torn supraspinatus tendon and retear after arthroscopic repair of full-thickness rotator cuff tears using the suture bridge technique. Am J Sports Med. 2023;51(9):2411-2421. 37345285 10.1177/03635465231178294

[72] Viechtbauer W. Conducting meta-analyses in R with the metafor package. J Stat Softw. 2010;36:1-48.

[73] Yeom JW Kholinne E Kim DM , et al. Postoperative HbA1c level as a predictor of rotator cuff integrity after arthroscopic rotator cuff repair in patients with type 2 diabetes. Orthop J Sports Med. 2023;11(2). 10.1177/23259671221145987 PMC9940196 36814763

[74] Zhang AL Montgomery SR Ngo SS , et al. Analysis of rotator cuff repair trends in a large private insurance population. Arthroscopy. 2013;29(4):623-629. 23375667 10.1016/j.arthro.2012.11.004

