# Supplementary material for: Prognostic Factors for Repair Integrity After Arthroscopic Rotator Cuff Repair: A Systematic Review and Meta-analysis
Source: Orthop J Sports Med. 2026 Jul 8;14(7):23259671261455856. doi: 10.1177/23259671261455856 (PMC13346710; doi:10.1177/23259671261455856)
Supplement: sj-pdf-1-ojs-10.1177_23259671261455856 – Supplemental material for Prognostic Factors for Repair Integrity After Arthroscopic Rotator Cuff Repair: A Systematic Review and Meta-analysis [file sj-pdf-1-ojs-10.1177_23259671261455856.pdf]

Prognostic factors for repair integrity after arthroscopic rotator cuff repair: a systematic review and meta-analysis

Supplementary material

Table of contents

SUPPLEMENTARY FILE 1: PRISMA CHECKLIST 2

SUPPLEMENTARY FILE 2: SEARCH STRATEGIES 4

EMBASE.COM 4
MEDLINE (OVID) 4
SCOPUS 4

SUPPLEMENTARY FILE 3: EXTRACTED DATA ITEMS 6

SUPPLEMENTARY FILE 4: CHALLENGES AND DETAILED PROCESSES IN CONDUCTING META-ANALYSES OF PROGNOSTIC FACTORS ESTIMATES 7

SUPPLEMENTARY FIGURE 1: PRISMA FLOWCHART (1) 8

SUPPLEMENTARY TABLE 1: DETAILED CHARACTERISTICS OF INCLUDED STUDIES 9

SUPPLEMENTARY FIGURES 2: FOREST PLOT OF INDIVIDUAL PROGNOSTIC FACTOR ESTIMATES 18

FIGURE 2A: ASSOCIATION BETWEEN INCREASING CORTICOSTEROID INJECTION AND REPAIR INTEGRITY 19
FIGURE 2B: ASSOCIATION BETWEEN INCREASED AGE AND REPAIR INTEGRITY 20
FIGURE 2C: ASSOCIATION BETWEEN INCREASING BODY MASS INDEX AND REPAIR INTEGRITY 21
FIGURE 2D: ASSOCIATION BETWEEN PRESENCE OF DIABETES AND REPAIR INTEGRITY 22
FIGURE 2E: ASSOCIATION BETWEEN FEMALE SEX AND REPAIR INTEGRITY 23
FIGURE 2F: ASSOCIATION BETWEEN INCREASING ACROMIOHUMERAL DISTANCE AND REPAIR INTEGRITY 24
FIGURE 2G: ASSOCIATION BETWEEN INCREASING FATTY INFILTRATION OF THE INFRASPINATUS AND REPAIR INTEGRITY 25
FIGURE 2H: ASSOCIATION BETWEEN INCREASING FATTY INFILTRATION OF THE SUPRASPINATUS AND REPAIR INTEGRITY 26
FIGURE 2I: ASSOCIATION BETWEEN INCREASING FATTY INFILTRATION AND REPAIR INTEGRITY 27
FIGURE 2J: ASSOCIATION BETWEEN INCREASING GLENOIDAL DISTANCE AND REPAIR INTEGRITY 28
FIGURE 2K: ASSOCIATION BETWEEN INCREASING MUSCLE ATROPHY OF THE SUPRASPINATUS AND REPAIR INTEGRITY 29
FIGURE 2L: ASSOCIATION BETWEEN INCREASING TEAR RETRACTION AND REPAIR INTEGRITY 30
FIGURE 2M: ASSOCIATION BETWEEN HIGHER DEGREE OF TEAR SEVERITY AND REPAIR INTEGRITY 31
FIGURE 2N: ASSOCIATION BETWEEN LARGER TEAR SIZE AND REPAIR INTEGRITY 32
FIGURE 2O: ASSOCIATION BETWEEN HIGHER DEGREE OF TENDON DEGENERATION AND REPAIR INTEGRITY 33

SUPPLEMENTARY TABLE 3: RISK OF BIAS OF INCLUDED STUDIES ACCORDING TO QUIPS TOOL 34

SUPPLEMENTARY TABLE 4: QUALITY OF SYNTHESIZED EVIDENCE REGARDING PROGNOSTIC FACTORS ESTIMATES 35

BIBLIOGRAPHY 36

Supplementary File 1: PRISMA checklist

Section and Topic	Item #	Checklist item	Location where item is reported
**TITLE**			
Title	1	Identify the report as a systematic review.	P.1
**ABSTRACT**			
Abstract	2	See the PRISMA 2020 for Abstracts checklist.	P.3
**INTRODUCTION**			
Rationale	3	Describe the rationale for the review in the context of existing knowledge.	P.5
Objectives	4	Provide an explicit statement of the objective(s) or question(s) the review addresses.	P.5
**METHODS**			
Eligibility criteria	5	Specify the inclusion and exclusion criteria for the review and how studies were grouped for the syntheses.	P.5
Information sources	6	Specify all databases, registers, websites, organisations, reference lists and other sources searched or consulted to identify studies. Specify the date when each source was last searched or consulted.	P.6
Search strategy	7	Present the full search strategies for all databases, registers and websites, including any filters and limits used.	P.6
Selection process	8	Specify the methods used to decide whether a study met the inclusion criteria of the review, including how many reviewers screened each record and each report retrieved, whether they worked independently, and if applicable, details of automation tools used in the process.	P.6
Data collection process	9	Specify the methods used to collect data from reports, including how many reviewers collected data from each report, whether they worked independently, any processes for obtaining or confirming data from study investigators, and if applicable, details of automation tools used in the process.	P.6
Data items	10a	List and define all outcomes for which data were sought. Specify whether all results that were compatible with each outcome domain in each study were sought (e.g. for all measures, time points, analyses), and if not, the methods used to decide which results to collect.	P.6
	10b	List and define all other variables for which data were sought (e.g. participant and intervention characteristics, funding sources). Describe any assumptions made about any missing or unclear information.	P.6
Study risk of bias assessment	11	Specify the methods used to assess risk of bias in the included studies, including details of the tool(s) used, how many reviewers assessed each study and whether they worked independently, and if applicable, details of automation tools used in the process.	P.6
Effect measures	12	Specify for each outcome the effect measure(s) (e.g. risk ratio, mean difference) used in the synthesis or presentation of results.	P.7
Synthesis methods	13a	Describe the processes used to decide which studies were eligible for each synthesis (e.g. tabulating the study intervention characteristics and comparing against the planned groups for each synthesis (item #5)).	P.7
	13b	Describe any methods required to prepare the data for presentation or synthesis, such as handling of missing summary statistics, or data conversions.	P.7
	13c	Describe any methods used to tabulate or visually display results of individual studies and syntheses.	P.7
	13d	Describe any methods used to synthesize results and provide a rationale for the choice(s). If meta-analysis was performed, describe the model(s), method(s) to identify the presence and extent of statistical heterogeneity, and software package(s) used.	P.8-9
	13e	Describe any methods used to explore possible causes of heterogeneity among study results (e.g. subgroup analysis, meta-regression).	P.9-10
	13f	Describe any sensitivity analyses conducted to assess robustness of the synthesized results.	P.10
Reporting bias assessment	14	Describe any methods used to assess risk of bias due to missing results in a synthesis (arising from reporting biases).	P.6
Certainty assessment	15	Describe any methods used to assess certainty (or confidence) in the body of evidence for an outcome.	P.7
**RESULTS**			
Study selection	16a	Describe the results of the search and selection process, from the number of records identified in the search to the number of studies included in the review, ideally using a flow diagram.	P.11
	16b	Cite studies that might appear to meet the inclusion criteria, but which were excluded, and explain why they were excluded.	P.11
Study	17	Cite each included study and present its characteristics.	P.11-13

Section and Topic	Item #	Checklist item	Location where item is reported
characteristics			
Risk of bias in studies	18	Present assessments of risk of bias for each included study.	P.16
Results of individual studies	19	For all outcomes, present, for each study: (a) summary statistics for each group (where appropriate) and (b) an effect estimate and its precision (e.g. confidence/credible interval), ideally using structured tables or plots.	P.13
Results of syntheses	20a	For each synthesis, briefly summarise the characteristics and risk of bias among contributing studies.	P.12
	20b	Present results of all statistical syntheses conducted. If meta-analysis was done, present for each the summary estimate and its precision (e.g. confidence/credible interval) and measures of statistical heterogeneity. If comparing groups, describe the direction of the effect.	P.13-16
	20c	Present results of all investigations of possible causes of heterogeneity among study results.	/
	20d	Present results of all sensitivity analyses conducted to assess the robustness of the synthesized results.	P.14-16
Reporting biases	21	Present assessments of risk of bias due to missing results (arising from reporting biases) for each synthesis assessed.	/
Certainty of evidence	22	Present assessments of certainty (or confidence) in the body of evidence for each outcome assessed.	P.16
**DISCUSSION**			
Discussion	23a	Provide a general interpretation of the results in the context of other evidence.	P.17-19
	23b	Discuss any limitations of the evidence included in the review.	P.20
	23c	Discuss any limitations of the review processes used.	P.20
	23d	Discuss implications of the results for practice, policy, and future research.	P.20
**OTHER INFORMATION**			
Registration and protocol	24a	Provide registration information for the review, including register name and registration number, or state that the review was not registered.	P.6
	24b	Indicate where the review protocol can be accessed, or state that a protocol was not prepared.	P.6
	24c	Describe and explain any amendments to information provided at registration or in the protocol.	/
Support	25	Describe sources of financial or non-financial support for the review, and the role of the funders or sponsors in the review.	P.21
Competing interests	26	Declare any competing interests of review authors.	P.21
Availability of data, code and other materials	27	Report which of the following are publicly available and where they can be found: template data collection forms; data extracted from included studies; data used for all analyses; analytic code; any other materials used in the review.	P.21

Supplementary File 2: Search strategies

Embase.com

('rotator cuff injury'/de OR 'rotator cuff rupture'/de OR 'rotator cuff repair'/de OR 'arthroscopic rotator cuff repair'/de OR 'supraspinatus tear'/de OR 'supraspinatus tendon tear'/de OR 'supraspinatus tendon rupture'/de OR 'supraspinatus tendinopathy'/de OR 'subscapularis tear'/de OR 'subscapularis tendon tear'/de OR 'subscapularis tendon rupture'/de OR (((cuff OR subscapularis OR supraspinatus OR infraspinatus OR 'teres minor') NEAR/4 (disease OR syndrome OR disorder* OR injur* OR lesion* OR tear* OR torn OR rupture* OR patholog* OR tendinopath* OR degeneration)) OR (('rotator cuff' OR subscapularis OR supraspinatus OR infraspinatus OR 'teres minor') NEAR/3 (surgery OR surgical OR repair* OR operation* OR operative OR reconstruct*))):ab,ti OR (('rotator cuff'/exp OR 'subscapularis muscle'/de OR 'subscapularis tendon'/de OR 'supraspinatus muscle'/de OR 'supraspinatus muscle tendon'/de OR 'supraspinatus tendon'/de OR 'infraspinatus muscle'/de OR 'infraspinatus tendon'/de) AND (disease OR syndrome OR disorder* OR injur* OR lesion* OR tear* OR torn OR rupture* OR patholog* OR tendinopath* OR degeneration OR surgery OR surgical OR repair* OR operation* OR operative OR reconstruct*):ab,ti))

AND

('surgery'/de OR 'arthroscopic surgery'/de OR 'tendon surgery'/de OR 'shoulder surgery'/de OR 'rotator cuff repair'/de OR 'arthroscopic rotator cuff repair'/de OR (surgery OR surgical OR repair* OR operation* OR operative OR reconstruct*):ab,ti)

AND

(2014:py OR 2015:py OR 2016:py OR 2017:py OR 2018:py OR 2019:py OR 2020:py OR 2021:py OR 2022:py OR 2023:py OR 2024:py)

NOT

(('animal'/de OR 'animal experiment'/exp OR 'nonhuman'/de) NOT ('human'/exp OR 'human experiment'/de))

NOT

[conference abstract]/lim

Medline (Ovid)

(rotator cuff injuries/ OR (((cuff OR subscapularis OR supraspinatus OR infraspinatus OR teres minor) ADJ4 (disease OR syndrome OR disorder* OR injur* OR lesion* OR tear* OR torn OR rupture* OR patholog* OR tendinopath* OR degeneration)) OR ((rotator cuff OR subscapularis OR supraspinatus OR infraspinatus OR teres minor) ADJ3 (surgery OR surgical OR repair* OR operation* OR operative OR reconstruct*))).ab,ti. OR (rotator cuff/ AND (disease OR syndrome OR disorder* OR injur* OR lesion* OR tear* OR torn OR rupture* OR patholog* OR tendinopath* OR degeneration OR surgery OR surgical OR repair* OR operation* OR operative OR reconstruct*).ab,ti.))

AND

(surgical procedures, operative/ OR arthroscopy/ OR general surgery/ OR (surgery OR surgical OR repair* OR operation* OR operative OR reconstruct*).ab,ti.)

AND

("2014" or "2015" or "2016" or "2017" or "2018" or "2019" or "2020" or "2021" or "2022" or "2023" or "2024").yr.

NOT

(exp animals/ NOT humans/)

Scopus

TITLE-ABS ((((cuff OR subscapularis OR supraspinatus OR infraspinatus OR "teres minor") W/4 (disease OR syndrome OR disorder* OR injur* OR lesion* OR tear* OR torn OR rupture* OR patholog* OR tendinopath* OR degeneration)) OR (("rotator cuff" OR subscapularis OR supraspinatus OR infraspinatus OR "teres minor") W/2 (surgery OR surgical OR repair* OR operation* OR operative OR reconstruct*)))

AND

(surgery OR surgical OR repair* OR operation* OR operative OR reconstruct*))

AND

(LIMIT-TO (PUBYEAR , 2021) OR LIMIT-TO (PUBYEAR , 2020) OR LIMIT-TO (PUBYEAR , 2019) OR LIMIT-TO (PUBYEAR , 2018) OR LIMIT-TO (PUBYEAR , 2017) OR LIMIT-TO (PUBYEAR , 2016) OR LIMIT-TO (PUBYEAR ,

2015) OR LIMIT-TO (PUBYEAR , 2014) OR LIMIT-TO (PUBYEAR , 2022) OR LIMIT-TO (PUBYEAR , 2023) OR LIMIT-TO (PUBYEAR , 2024))

Supplementary File 3: Extracted data items

Item N°	Specification
**Study characteristics**	
1	Title, first author and year of publication
2	Country of research group
3	Research design (prospective vs retrospective)
**Participants**	
4	Inclusion criteria
5	Exclusion criteria
6	Number of included patients
7	Number of studied shoulders
8	Patients' average age
9	Patients' sex ratio
**Intervention**	
10	Surgical intervention definition
11	Rehabilitation protocol
**Follow-up**	
12	Duration of follow-up (minimum-maximum)
**Outcomes**	
13	Number of studied outcomes
**Per outcome**	
14	Targeted outcome type (repair integrity, shoulder function, shoulder stiffness)
15	Definition or scale used
16	Type (continuous, dichotomous, categorized, unclear)
17	Assessment time point (in months)
18	Blinding measurement (yes/no)
19	Analysis of risk factors (univariable vs multivariable)
**Per prognostic factor**	
20	Definition
21a	Type (continuous, dichotomous, categorized, unclear)
21b	Classes (in case of dichotomous or categorized)
23	Method (univariable, multivariable or both)
24a	Effect estimate type (result of a test, odds ratio, risk ratio, mean difference, coefficients)
24b	Effect estimate (e.g. odds ratio = 2.05)
24c	Effect estimate accuracy (e.g. odds ratio confidence interval 95% = [2.01 ; 2.09])
24d	p-value (e.g. p = 0.05)

.

Supplementary File 4: Challenges and detailed processes in conducting meta-analyses of prognostic factors estimates

Different challenges arise when conducting a meta-analysis of association measures, including a high variability in the reporting of: (a) association measures (regression coefficients, odds ratios, risk ratios, etc…), (b) outcome description and type (dichotomized, continuous, categorized), (c) prognostic factor description and type (dichotomized, continuous, categorized), (d) direction of association at the outcome and prognostic factor levels, two examples illustrate this: first, at the outcome level: one study reported prognostic factors associated with an intact tendon, while another reported prognostic factors associated with a tendon defect; second, at the prognostic factor level: one study used male biological sex as the reference category in their multivariable model, while another used female sex as the reference; and lastly (e) set of variables included in the final multivariable model.

These challenges necessitate: (1) a standardization step across studies at the outcome and prognostic factor level, (2) a step of converting association measures into standardized metrics, (3) a final step of pooling these standardized measures in a meta-analysis.

Standardizing reporting across studies at the outcome and prognostic factor levels

All reported prognostic factors in the included studies were considered in this standardization step. For each prognostic factor, we defined a canonical direction (e.g., "higher age is associated with…") and adjusted the reported estimates accordingly. In some cases, this required inverting the reported outcome prevalence or odds ratio (OR) and the associated 95% confidence interval (CI). Individual prognostic factors were grouped under broader, similar concepts (e.g., tear size encompassed anteroposterior, mediolateral, and tear size areas). For each prognostic factor, the direction of association was then reported (e.g. "positive association: when tear size increases, the risk of defect increases"). Whenever needed, regression coefficients were converted to odds ratios. This standardization process was initially performed by two study authors ([redacted]) and independently reviewed by two others ([redacted]).

Supplementary Figure 1: PRISMA Flowchart (1)

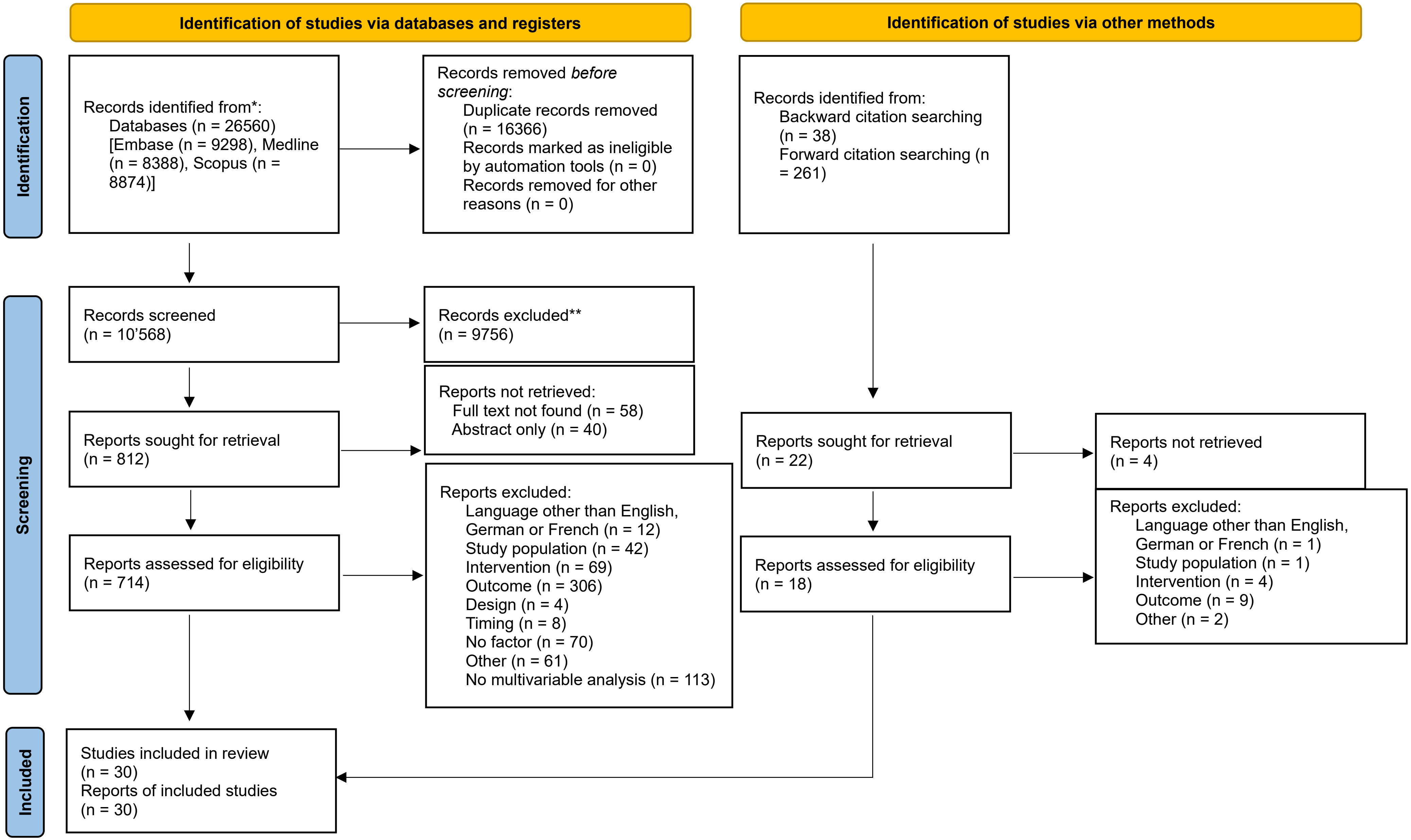

*Consider, if feasible to do so, reporting the number of records identified from each database or register searched (rather than the total number across all databases/registers).
**If automation tools were used, indicate how many records were excluded by a human and how many were excluded by automation tools.

Supplementary Table 1: Detailed characteristics of included studies

Author	Year	Design	Participants	Intervention	Rehabilitation protocol	Definition	Classes	Value	Timepoint	Statistical analysis method
**Park, J.S.**[56]	2015	Prospective	Patients with degenerative and traumatic injuries, full-thickness small and medium-sized (<3 cm) supraspinatus, infraspinatus or subscapularis tears, had pre-operative MRI, intra-operative arthroscopic evaluation, and MRI or CTA at least 1 year after surgery, between March 2004 and August 2012; Exclusion of patients with previous shoulder surgery, isolated subscapularis tear, glenohumeral arthritis	One surgeon involved, single-row or double-row repair, biceps procedure (debridement, tenotomy, tenodesis), distal clavicle resection	Post-operative immobilization for 4 weeks for small tear, 5 weeks for medium sized tears, at week 6 beginning of active assisted ROM, muscle strengthening exercises after full passive ROM obtained and sport activities allowed 6 months after surgery	Image based evaluation with MRI or CTA at 1-year follow-up, evaluated by a musculoskeletal radiologist, used of Sugaya et al. classification for MRI	Defect (predicted): on CTA, contrast media leakage through the footprint with discontinuity of the rotator cuff insertion from the footprint (stage III) or on MRI, according to the classification of Sugaya et al. types IV and V - Intact: on CTA stage I, II or on MRI, types I to III	45/339	12	Multivariate logistic regression with a forward stepwise method using statistically significant factors in the univariate analysis
**Tan, M.**[69]	2016	Retrospective	Patients with degenerative and traumatic supraspinatus tears, primary repair between June 2005 and July 2013; Exclusion of patients with revision surgeries, repairs using a polytetrafluoroethylene patch, repairs for glenohumeral arthritis or patients enable to attend the 6 months follow-up	One surgeon involved, single-row arthroscopic knotless inverted mattress suture tension band performed	No immobilization described, between week4 to 8 after surgery passive shoulder flexion-extension, external-internal rotation and abduction exercises, active shoulder movement and isometric strengthening at week 6, exercises with a TheraBand, overhead activities and lifting up to 10 kg after 3 months	Image based evaluation with ultrasound at 6-month follow-up, comparison with the pre-operative images by the same sonographer (no classification mentioned)	Defect (predicted): full- or partial-thickness defects in the rotator cuff tendon that were smaller or larger than the original tear - Intact: no tear observed after surgery	176/1300	6	Multivariate logistic regression with 8 variables chosen by the authors
**Tashijan, R.Z.**[70]	2016	Retrospective	Patients with a supraspinatus tear, etiology not precisely described and already included in an ongoing genetic study (not named), no period mentioned; Exclusion of patients with revision surgeries, partial repairs, repairs involving margin convergence or with less than 1 year follow-up after surgical repair	One surgeon involved, single-row repair using triple-loaded suture anchors and simple stitches, double-row repairs with a suture bridge construct	Post-operative immobilization in a sling for 6 weeks, for small and medium sized tears, passive supine forward elevation in the scapular plane to 130° and external rotation at the side to 30° at week 2 postoperatively and for large and massive sized tears, pendulums for 6 weeks, after 6 weeks and for all patients, passive and active assisted motion in all planes, except internal rotation and extension and isometric and resistive shoulder strengthening exercises and internal rotation and extension stretching after 3 months	Image based evaluation with MRI at 1-year follow-up, used of pattern defined by Cho et al.	Defect (predicted): type I lateral retears of Cho et al. pattern, no remaining cuff tissue at the insertion site of the rotator cuff on the greater tuberosity and type II medial retears of Cho et al. pattern, remnant of cuff tissue remained at the insertion site despite the retear - Intact: presence of continuous tissue from the lateral tendon edge extending to the greater tuberosity on every MRI section	30/72	12	Multivariate regression with variables chosen by the authors

Author	Year	Design	Participants	Intervention	Rehabilitation protocol	Definition	Classes	Value	Timepoint	Statistical analysis method
							(coronal and sagittal)			
**Diebold, G.[16]**	2017	Retrospective	Patients with all types of rotator cuff tears, etiology not precisely described, between February 2004 and December 2014; Exclusion of patients with revision procedure, concurrent surgery at the time of the rotator cuff repair, repaired with a synthetic parch, irreparable tear or partially repaired or unperformed ultrasound at 6 months follow-up	One surgeon involved, arthroscopic single-row knotless inverted mattress construct	Two types of rehabilitation protocols depending on the time of the surgery (not precisely described), for earlier years, immediate passive range-of-motion exercises and active at day 8 post-operative, increased intensity of active exercises at week 6, active resistance after 3 months - for later years, 6 weeks immobilization with an abduction sling, ROM exercises at day 8 post-operative and gradually increased, isometric exercises at week 6, active resistance introduced after 3 months	Image based evaluation with ultrasound at 6-month follow-up, evaluated by experienced musculoskeletal sonographers (no classification mentioned)	Defect (predicted): any defect, either full or partial-thickness, visible on ultrasound, irrespective of its size - Intact: without defect on ultrasound	212/1600	6	Multivariate logistic regression with all the variables described in the paper chose by the authors, elaboration of a predictive equation logit (p)
**Kang, Y.[35]**	2017	Retrospective	Patients with chronic stage of a full-thickness tear of the supraspinatus determined pre-operatively with MRA and confirmed intra-operatively, performed MRI at 1 year follow-up, between January and December 2013; Exclusion of patients with poor image quality on pre-operative MRA or Sugaya type III or IV on the follow-up MRI	Number of surgeons not precisely described, single or double-row procedures	Rehabilitation protocol not precisely described	Image based evaluation with MRI at 1-year follow-up, two independent evaluations by two radiologists (one senior and one junior) using Sugaya et al. classification	Defect (predicted): type V Sugaya et al. classification - Intact: type I and III	20/50	12	Binary logistic regression with a forward stepwise conditional method with variables chosen by the authors
**Rashid, M.S.[58]**	2017	Retrospective	Patients with a full-thickness degenerative supraspinatus tears, more than 50 years old, radiologically confirmed, between November 2007 and February 2012; Exclusion of patients with irreparable tears, no complete repair, or no MRI during follow-up	65 surgeons at 47 centers, arthroscopic rotator cuff repair or mini-open surgery, not precisely described	Rehabilitation protocol not precisely described	Image based evaluation with MRI or CTA at 1-year follow-up, evaluation by one senior musculoskeletal radiologist (no classification mentioned)	Defect (predicted): a full-thickness defects of the supraspinatus tendon - Intact: the supraspinatus tendon remained attached to the proximal humerus or a partial-thickness defects when low grade	95/220	12	Multivariate logistic regression with variables chosen by the authors
**Baverel, L.[5]**	2018	Retrospective	Patients with degenerative and traumatic full-thickness supraspinatus tears, repaired by double-row suture technique, complete clinical and ultrasound evaluations at least 1 year after surgery, between January 2007 and June 2010; Exclusion of	One surgeon involved, double-row and number of anchors depending on the tear size (not precisely described)	Post-operative immobilization in a 20° abduction sling for 6 weeks with passive motion exercises, active shoulder motion after 6 weeks, active passive motion was started earlier according to the	Image based evaluation with ultrasound at 1-year follow-up, evaluated by an ultrasound radiologist using adapted Sugaya et al. classification for CTA	Defect (predicted): types IV and V Sugaya et al. classification - Intact: types I to III	27/212	12	Multivariate logistic regression with 10 variables chosen by the authors

Author	Year	Design	Participants	Intervention	Rehabilitation protocol	Definition	Classes	Value	Timepoint	Statistical analysis method
			patients with revision cases, concomitant surgery on the ipsilateral shoulder at the same time or Hamada stage >2		preoperative tear size and light sports and demanding activities were allowed after 6 months					
**Kim, Y.K.**[40]	2018	Retrospective	Patients with degenerative and traumatic injuries, all types of full-thickness medium-sized or larger rotator cuff tears, had post-operative MRI at 2 years after surgery, between February 2007 and August 2014; Exclusion of patients with incompletely repaired tendons, irreparable rotator cuff tear or isolated tears of supraspinatus	One surgeon involved, single-row and double-row suture-bridge were performed, double-loaded anchors	Post-operative immobilization immediately after surgery, pendulum exercise and deltoid isometric exercise at week 1 post-operative, passive range of motion stretching exercises after 3 weeks and active range of motion exercises after 6 weeks	Image based evaluation with MRI at 2-year follow-up, used of Sugaya et al. classification, no clear definition provided	Defect (predicted): types IV and V Sugaya et al. classification - Intact: types I to III	28/180	24	Multivariate binary logistic regression using a backward elimination method with variables chosen by the authors
**Liu, X.N.**[45]	2018	Retrospective	Patients with degenerative and traumatic injuries, symptomatic 2-4 cm size full-thickness supraspinatus tear confirmed by pre-operative MRI, repaired with arthroscopic transosseous suture, performed an anatomic outcome analysis at 1 year after surgery with MRI and a functional outcome analysis at 2 years after surgery, between May 1, 2013 and August 31, 2014; Exclusion of patients with previous shoulder surgery, isolated supraspinatus tear, partial or small tear and large or massive tear or glenohumeral arthritis	One surgeon involved, arthroscopic TOS rotator cuff repair for superior and/or posterosuperior lesions, 2-tunnel technique for intersecting TOS: suture passage using a combined antegrade/retrograde delivery technique, with retrieval and tying by either a simple stitch or an X-BOX stitch configuration (randomly allocated prior to surgery by assignment of the randomization number to the subjects), side-to-side sutures for L-shaped/reverse L-shaped tears and margin convergence techniques for larger U-shaped tears, were used	Post-operative immobilization for 6 weeks, pendulum exercises after week 1, start of active assisted range of motion exercises after 6 weeks	Image based evaluation with MRI at 1-year follow-up, evaluated by a musculoskeletal radiologist using Sugaya et al. classification	Defect (predicted): types III to V Sugaya et al. classification - Intact: types I and II	9/27	12	Multivariate logistic regression using statistically significant factors from the univariate analysis after the multicollinearity test
**Herring, M.J.**[30]	2019	Retrospective	Patients with all types of primary rotator cuff tears, etiology not precisely described, > 18 years old, pre-operative WORC questionnaire collection, between 2009 and 2013; Exclusion of patients with conversion to open repair, uncompleted repair or failed of 2 years follow-up	One surgeon involved, arthroscopic rotator cuff repair, subacromial decompression, distal clavicle excision, biceps tenodesis or tenotomy, and/or labral debridement at the discretion of the treating surgeon	Post-operative immobilization for 6 weeks, physical therapy performed at home, after immobilization started on formal outpatient physical therapy with passive motion initially with graduation to active motion and strengthening (time not precisely described)	Symptomatic evaluation after a minimum of 2-year follow-up with or without persistent or recurrent shoulder pain and/or weakness (evaluation of symptoms not precisely described) and, image-based evaluation with MRI only if there is persistent pain (no classification mentioned)	Defect (predicted): patients with symptoms and a retear in MRI - Intact: patients without symptoms or patients with symptoms but without retear on MRI	28/500	24	Multivariate logistic regression, unclear set of variables

Author	Year	Design	Participants	Intervention	Rehabilitation protocol	Definition	Classes	Value	Timepoint	Statistical analysis method
**Kwon, J.**[41]	2019	Retrospective	Patients with a full-thickness or high-grade partial-thickness subscapularis or infraspinatus tears, etiology not precisely described, had pre-operative MRI and MRI or CTA at least 1-year post-operative, between February 2010 and December 2014; Exclusion of patients with previous surgery on the same shoulder, irreparable rotator cuff tears or isolated subscapularis tears	One surgeon involved, anterosuperior portal via the rotator interval to manage the intra-articular lesions, the posterior and anterior portals were moved to the subacromial space, subacromial decompression to remove the inflamed bursal tissues, single row (large to massive tears) or double row technique (small tears) with the modified Mason Allen technique, suture bridge technique (medium-and large-sized tears), self-locking sliding knots	Post-operative immobilization for 4 weeks for partial-thickness and small tears, for 5 weeks for medium tears or for 6 weeks for large or massive tears, active ROM exercises were initiated after weaning patients from the braces, muscle strengthening after 6 to 12 weeks post-operative	Image based evaluation with MRI or CTA at 1-year follow-up, evaluated by a musculoskeletal radiologist with 10 years of experience and the surgeon to reach a consensus using Sugaya et al. classification for MRI or evidence of contrast leakage for CTA	Defect (predicted): types IV and V Sugaya et al. classification on MRI or evidence of contrast leakage on CTA - Intact: types I to III on MRI or no contrast on CTA	101/531	12	Multivariate logistic regression with a stepwise forward conditional method with variables chosen by the authors
**McColl, A.H.**[49]	2019	Retrospective	Patients with all types of primary rotator cuff tears, etiology not precisely described, with an arthroscopic knotless single-row inverted mattress technique, performed ultrasound at 6 month post-operative, between October 2005 and October 2013; Exclusion of patients with revision surgery, shoulder arthroplasty, fracture associated with rotator cuff repairs, used of polytetrafluoroethylene patches, irreparable rotator cuff tears, partially repaired or isolated subscapularis repair	One surgeon involved, single-row knotless inverted mattress technique with bursal or an undersurface approach, partial thickness tears were converted into full thickness tears before reduction	Two types of rehabilitation protocols depending on the time of the surgery (not precisely described), for early years, immediate passive range-of-motion exercises, active ROM exercises at day 8, increased intensity of active ROM after 6 weeks and until 3 months post-operative - for later years, use of a sling to immobilize the arm for 6 weeks, passive ROM after 6 weeks, isometric strengthening exercises and resistance exercises after 3 months	Image based evaluation with ultrasound at 6-month follow-up, evaluated by ultrasonographer using a standardized method	Defect (predicted): full thickness defect in the rotator cuff tissue detected on ultrasound examination - Intact: no thickness on ultrasound	240/1600	6	Multivariate logistic regression with a stepwise backward regression analysis
**Park, S.G.**[57]	2019	Retrospective	Patients with degenerative and traumatic injuries, all types of large to massive full-thickness tears confirmed by pre-operative MRI and using a 5-mm premarked probe at the time of surgery, not amenable to complete repair with meticulous release of fibrous bursal and articular adhesions, had at least 1 year follow-up with MRI and functional outcome assessment, between January 2015 to September 2017; Exclusion of patients with previous surgery on the shoulder, incomplete or partial repair, isolated subscapularis tears,	One surgeon involved, suture-bridge technique in the lateral decubitus position, four routine arthroscopic portals (anterior, posterior, lateral, anterolateral), tenotomy or tenodesis if needed, single-row fashion or a mattress repair with transtendon technique, acromioplasty, knotless anchors	Post-operative immobilization for 6 weeks with the arms placed in an abduction brace positioned in 30° abduction, pendulum exercises started at day 3, gentle, self-assisted, passive ROM exercises at week 6, isotonic strengthening exercises after 3 months	Image based evaluation with MRI at a minimum of 1-year follow-up, evaluated by 2 independent musculoskeletal radiologists using Sugaya et al. classification	Defect (predicted): types IV and V Sugaya et al. classification - Intact: types I to III Sugaya classification	19/50	12	Multivariate logistic regression with a stepwise forward conditional method

Author	Year	Design	Participants	Intervention	Rehabilitation protocol	Definition	Classes	Value	Timepoint	Statistical analysis method
			glenohumeral arthritis or inflammatory arthropathy							
**Chen, Y.[8]**	2020	Prospective	Patients with degenerative and traumatic injuries, all types of small- to large-sized full-thickness tears confirmed by pre-operative MRI, complete repair, had at least 2 years follow-up, between November 2015 and May 2017; Exclusion of patients with bilateral surgery or previous surgery on shoulder, concomitant distal clavicle resection, shoulder instability, fractures of the shoulder, glenohumeral osteoarthritis greater than grade 2, begin shoulder activities before 6 months postop	One senior surgeon involved, biceps tendon (debridement, tenodesis or tenotomy) if needed, subacromial bursectomy and acromioplasty, according to the tear size and shape (not precisely described), rotator cuff repaired with suture anchors using a single-row or transosseous-equivalent (suture-bridge) double-row technique	Post-operative immobilization for 6 weeks in an abduction sling, passive shoulder flexion, external rotation, isometric strengthening exercises at day 2, active motion initiated after 6 months	Image based evaluation with MRI at a minimum of 24-month follow-up, evaluated by an experienced radiologist using Sugaya et al. classification	Defect (predicted): types IV and V Sugaya et al. classification - Intact: types I to III	24/145	24	Multivariate logistic regression based on a threshold (P < .1) in the univariate analysis
**Choi, S.[10]**	2014	Retrospective	Patients with all types of full-thickness rotator cuff tears confirmed by pre-operative MRI, failed initial non-operative treatment, with single or multiple tendon involvement, between May 2008 and September 2010; Exclusion of patients with revision surgery, small tears size, labral tears, glenohumeral arthritis, inflammatory diseases or no MRI during follow-up	One surgeon involved, double row rotator cuff repair performed in a semilateral position, subacromial decompression, number of anchors depended on the tear size (not precisely described)	Post-operative immobilization in an abduction brace for 4 to 6 weeks, pendulum exercises after the day of the surgery, passive forward flexion exercises after 1 week, active exercises after 6 weeks	Image based evaluation with MRI at a minimum of 1-year follow-up, used od Sugaya et al. classification and pattern defined by Cho et al.	Defect (predicted): types IV and V Sugaya et al. classification - Intact: types I to III	25/147	12	Multivariate logistic regression, unclear set of variables
**Le, B.T.[43]**	2014	Retrospective	Patients with degenerative and traumatic injuries, all types of primary rotator cuff tears, repaired with an arthroscopic knotless inverted-mattress technique, had evaluation by ultrasound at 6 months post-operative, between January 2006 and June 2011; Exclusion of patients with previous rotator cuff repair on the same shoulder, no repaired or incompletely repair or used of synthetic polytetrafluoroethylene patch	One surgeon involved, arthroscopic surgery of the glenohumeral joint through a posterior portal, knotless inverted-mattress fixation technique with a single row of sutures, partial thickness tears converted into full-thickness tears before repair	Post-operative immobilization for 6 weeks, gentle passive range of motion exercises after 6 weeks, between 6 weeks and 3 months, isometric strengthening exercises and between 3 and 6 months, active resistance exercises	Image based evaluation with ultrasonography at 6-month follow-up, evaluated by 1 for 2 experienced musculoskeletal sonographers (no classification mentioned)	Defect (predicted): any full- or partial-thickness defect visible on ultrasound, irrespective of size - Intact: no thickness on ultrasound	174/1000	6	Multivariate logistic regression with all the variables described in the paper chosen by the authors, elaboration of a predictive equation logit (p)
**Lobo-Escolar, L.[46]**	2020	Retrospective	Patients with degenerative and traumatic injuries, a full-thickness supraspinatus tear, with or without a combined tear of other rotator cuff tendon, between January 2008 and December 2010; Exclusion of patients with revision surgery, irreparable tear, isolated subscapularis injury, margin	Number of surgeon not precisely described, conservative treatment procedure at first and if it failed after 3 months, surgery with full thickness rotator cuff tear smaller than 1 cm repaired by single-row and for 1cm or larger tears, double-row	Post-operative immobilization in an abduction pillow for 3 weeks, pendulum exercises and passive motion during 4 weeks, active assisted motion with pulley at week 6, active range of motion between 7-8 weeks, strengthening exercises after 10-12 weeks	Combined image based evaluation with MRI using Sugaya et al. classification, and symptomatic evaluation using visual analogue scale score for pain and Jobe test and/or external rotation lag	Defect (predicted): symptomatic tear with intensive pain (VAS score >=5) and/or functional impairment (positive Jobe test and/or external rotation lag sign) combined with	15/158	6	Multivariate logistic regression with statistically significant differences between groups in the bivariant analysis (sex and age also included)

Author	Year	Design	Participants	Intervention	Rehabilitation protocol	Definition	Classes	Value	Timepoint	Statistical analysis method
			convergence rotator cuff repair or associated calcifying tendonitis	and suture bridge/transosseous equivalent were performed		sign for functional impairment	types IV or V Sugaya et al. classification - Intact: types I to III Sugaya et al. classification, with or without symptomatic tear			
**Duong, J.K.H.**[17]	2021	Retrospective	Patients with primary full-thickness rotator cuff tears or partial-thickness tears > 50% (tear pattern not precisely described), etiology not precisely described, had ultrasound at 6 months post-operative, between January 2007 and December 2018; Exclusion of patients with revision surgery, another shoulder procedure, partial repairs, irreparable tears, isolated subscapularis tears or used of a polytetrafluoroethylene patch	One surgeon involved in several hospitals, single-row rotator cuff repair, converting partial-thickness tears into full-thickness tears, knotless inverted-mattress fixation, undersurface approach or bursal approach	Two types of rehabilitation protocols depending on the time of the surgery (not precisely described), for earlier years, immediate passive range of motion exercises, active range of motion exercises after day 8 post-operative, increase of ROM at week 6, active resistance after 3 months - for later years, post-operative immobilization for 6 weeks, introduction of ROM exercises at day 8, isometric strengthening exercises at week 6, exercises against resistance after 3 months	Image based evaluation with ultrasonography at 6-month follow-up, evaluated by an experienced musculoskeletal sonographer using a standardized method	Defect (predicted): a full- or partial-thickness defect was visible as a hypoechoic gap on ultrasound, regardless of tear size - Intact: no thickness on ultrasound	271/1962	6	Multivariate logistic regression with all the variables described in the paper chosen by the authors, elaboration of a predictive equation logit (p)
**Lim, T.K.**[44]	2021	Retrospective	Patients with a symptomatic degenerative and traumatic injuries, symptomatic supraspinatus/infraspinatus and/or subscapularis tears, first shoulder surgery, had MRI at 6 months post-operative, between May 2011 and May 2018; Exclusion of patients with revision surgery, open repair, reverse shoulder arthroplasty, tendon transfer, superior capsular reconstruction, partial repair or isolated subscapularis tear	One surgeon involved, single row technique or suture bridge technique were performed	Post-operative immobilization for 4 weeks in an abduction brace with elbow and finger exercises, after the removal of the brace, stretching exercises of the shoulder joint, strengthening exercises after 3 months	Image based evaluation with MRI at 6-month follow-up, evaluated by an experienced musculoskeletal radiologist using Sugaya et al. classification	Defect (predicted): types IV and V Sugaya et al. classification - Intact: types I to III	26/200	6	Multivariate logistic regression with a stepwise forward conditional method
**Guo, A.A.**[26]	2022	Retrospective	Patients with degenerative and traumatic injuries, multiple tendon tears, primary surgery, retourned to clinical evaluation at 6 weeks post-operative and ultrasound evaluation at 6 months, between January 2005 and December 2020; Exclusion of patients with concurrent procedures (capsular release, stabilization, calcific tendinitis debridement or fracture reduction), irreparable or partially repaired tendons, used of an interpositional	One senior surgeon involved at public and private hospitals, patients in the upright beach-chair position, partial-thickness tears were converted into full-thickness tears, torn rotator cuff tendon was reattached to the greater tuberosity using a single row of suture anchors in a knotless, inverted-mattress configuration	Two types of rehabilitation protocols depending on the time of the surgery (not precisely described), for earlier years, passive range-of-motion exercises started immediately of the surgery, active range-of-motion exercises at day 8 post-operative, active exercises increased at week 6, active resistance after 3 months - for later years, post-operative immobilization for 6 weeks,	Image based evaluation with ultrasound at 6-month follow-up, evaluated by an experienced musculoskeletal sonographer (no classification mentioned)	Defect (predicted): not precisely described - Intact: not precisely described	145/1526	6	Multivariate logistic regression with statistically significant values in the univariate analysis, elaboration of a predictive equation logit (p)

Author	Year	Design	Participants	Intervention	Rehabilitation protocol	Definition	Classes	Value	Timepoint	Statistical analysis method
			polytetrafluoroethylene patch or failure to return for 6-week follow-up		range-of-motion exercises after 8 days, isometric strengthening exercises at week 6, resistance exercises after 3 months					
**Caffard, T.**[7]	2023	Retrospective	Patients with degenerative and traumatic injuries, a complete tear of supraspinatus, with a minimum of 2 years follow-up, between August 2012 and December 2015; Exclusion of patients with other treatment than double-row technique, partial rupture or missing MRI planes or slices	Two surgeons involved, double-row techniques, initially patients treated with nonoperative therapy at least 6 weeks before surgery	Post-operative immobilization in an abduction pillow for 6 weeks with passive exercises, strengthening exercises after 13 weeks	Image based evaluation with MRI at a minimum of 2-year follow-up, evaluated by 2 clinicians using Sugaya et al. classification	Defect (predicted): types IV and V Sugaya et al. classification - Intact: types I to III	6/55 (**assumption**)	24	Logarithmic multivariate regression with all the variables described in the paper chosen by the authors
**Erşen, A.**[18]	2023	Retrospective	Patients with degenerative and traumatic injuries, all types of full-thickness small to medium sized tears confirmed by pre-operative MRI, had post-operative MRI at least 9 months after surgery, > 18 years, between April 2016 and September 2020; Exclusion of patients with revision surgery, surgical technique other than double-row suture bridge, moderate or severe fatty infiltration of supraspinatus or infraspinatus muscles, severe retraction of the torn tendon	One surgeon involved, double-row technique, knotless suture-bridge, 1 anchor for small tears and 2 anchors for medium-sized tears	Post-operative immobilization for 4 weeks, started of active hand, wrist and elbow motion and passive shoulder motion right after the surgery, active assisted range of motion exercises at week 4, active range of motion, strengthening and proprioceptive exercises at week 8, sports and full physical activity after 6 months	Image based evaluation with MRI at 1-year follow-up, evaluated by a senior musculoskeletal radiologist using Sugaya et al. classification	Defect (predicted): types IV and V Sugaya et al. classification - Intact: types I to III	19/94	12	Multivariate logistic regression with statistically significant values in the univariate analysis
**Johnson, A.H.**[34]	2023	Retrospective	Patients with degenerative and traumatic injuries, all types of rotator cuff tears, surgery with arthroscopic double-row and 4 suture anchors, pre-operative MRI available, between February 1, 2024 and March 31, 2020; Exclusion of patients with open surgery	Seven surgeons involved, double row with 4 suture anchors, medial row anchors, lateral row fixation were used	Post-operative immobilization for 6 weeks, home exercise programme with hand, wrist and elbow range of motion and shoulder pendulums between the day after the surgery and 10-14 days after, passive shoulder range of motion at week 2, active assist range of motion at week 4-6, active range of motion after 6-8 weeks	Symptomatic evaluation (time not precisely described) and, only for symptomatic patients, confirmed by non-contrast MRI (no classification mentioned) or intra-operatively during a second surgery	Defect (predicted): symptomatic retears, not precisely described - Intact: no symptom	26/343	12	Multivariate logistic and linear regression based on knot fixation type at the medial row and by suture type after controlling for potentially confounding variables
**Kim, M.S.**[39]	2023	Retrospective	Patients with all types of rotator cuff tears, etiology not precisely described, diagnosed with diabetes mellitus, pre-operative and post-operative evaluation of serum glycosylated haemoglobin levels, had MRI at 6 months after surgery, between January 2016 and November 2019; Exclusion of patients with open surgery, massive tear after partial repair or lost to follow-up	One surgeon involved, arthroscopic double-row suture bridge RCR, acromioplasty was performed when needed, mattress sutures, suture limbs were used to form a suture bridge over the tendons, tenotomy or tenodesis was performed when needed	Post-operative immobilization for 6 weeks, active-assisted motion exercises at week 6, active assisted exercise 2 times a day, 10 rounds each time, muscle strengthening exercises at week 12, competitive sports activities after 6 months	Image based evaluation with MRI (time not precisely described), evaluated by two independent fellowship-trained shoulder orthopaedic surgeons using Sugaya et al. classification	Defect (predicted): types IV and V Sugaya et al. classification - Intact: types I to III	32/103	6	Backward stepwise multivariate logistic regression with statistically significant values in the univariate analysis

Author	Year	Design	Participants	Intervention	Rehabilitation protocol	Definition	Classes	Value	Timepoint	Statistical analysis method
**Manop, P.**[48]	2023	Retrospective	Patients with all types of full-thickness rotator cuff tears confirmed by arthroscopy and post-operative MRI at least 6 months after surgery, etiology not precisely described, between February 2012 and February 2021; Exclusion of patients with revision surgery or isolated subscapularis tear	One fellowship-trained sports medicine surgeon involved, biceps tenotomy or tenodesis, synovectomy, or capsular release were performed when needed, all arthroscopic repair techniques used the transosseous-equivalent repair techniques depending on the tear size and characteristics	Post-operative immobilization in an abduction sling for 4 to 6 weeks, active elbow, wrist, and hand motion immediately after the surgery, passive shoulder motion at week 2, active shoulder motion at week 6, return-to-sport activities after 6 months	Image based evaluation with MRI at a minimum of 6-month follow-up, evaluated by an orthopaedic surgeon and a musculoskeletal radiologist using Sugaya et al. classification	Defect (predicted): types IV and V Sugaya et al. classification - Intact: types I to III	27/133	6	Multivariate logistic regression, unclear set of variables
**Olthof, M.G.L.**[54]	2023	Retrospective	Patients with degenerative and traumatic injuries, a full-thickness supraspinatus tear with fatty infiltration of this muscle not more than Goutallier stage 2, between May 2014 and March 2017; Exclusion of patients with previous surgery on the shoulder, osteoarthritis of the glenohumeral joint, inflammatory joint diseases or use of oral steroids or immunosuppressive drugs	Number of surgeons not precisely described, arthroscopic rotator cuff repair, tenotomy, tenodesis, subacromial debridement were performed when needed, single or double-row repair was performed	Post-operative immobilization in a brace at 30° for 6 weeks, active full range of motion exercises at week 7-12, strengthening exercises after 13 weeks	Image based evaluation with MRI at 1-year follow-up, evaluated by a fellowship-trained musculoskeletal radiologist using Sugaya et al. classification	Defect (predicted): types IV and V Sugaya et al. classification - Intact: types I to III	21/97	12	Forward-conditional binomial multivariate logistic regression with all the variables described in the paper chosen by the authors
**Tokunaga, T.**[72]	2023	Retrospective	Patients with degenerative and traumatic injuries, all types of full-thickness tears, underwent intra-operative biopsy to assess the degree of histological degeneration, pre-operative MRI and at least 6 months after surgery, with 12 and 24 months follow-up consultations, SB-mediated arthroscopy, between April 2012 and July 2020; Exclusion of patients with revision surgery, partial repair, isolated subscapularis tear, coexistence of fractures, recurrent shoulder dislocation or Hamada grade 3	Three surgeons involved, long head of biceps treatment, synovectomy, and subacromial decompression were performed when needed, single-row or suture bridge repair	Post-operative immobilization in an abduction brace (time not precisely described), active motion of the elbow, wrist and hand at week 1 for small tears and week 3 for medium, large and massive tears, active range of motion exercises at week 6 muscle-strengthening exercises at week 12, heavy manual work and sports activity after 6 months	Image based evaluation with MRI at a minimum of 6-month follow-up, used of Sugaya et al. classification	Defect (predicted): types IV and V Sugaya et al. classification - Intact: types I to III	22/187	6	Multivariate logistic regression with statistically significant values in the univariate analysis
**Yeom, J.W.**[74]	2023	Retrospective	Patients with degenerative and traumatic injuries, medium to large complete tear of equal or more than 2 tendons and medial retraction of Patte grade 1 or 2 on preoperative MRI, having type 2 diabetes mellitus with pre-operative evaluation of HbA1c levels and at 6- and 12- months post-operative, between January 1, 2007 and December 31, 2019; Exclusion of patients with previous rotator cuff surgery,	One surgeon involved, single or double row performed, no clear other details	Rehabilitation protocol not precisely described	Image based evaluation with MRI at 1-year follow-up, evaluated by a fellowship trained shoulder surgeon using Sugaya et al. classification	Defect (predicted): types IV and V Sugaya et al. classification - Intact: types I to III	35/183	12	Multivariate logistic regression using variables with P values of >.20 in the univariate analysis

Author	Year	Design	Participants	Intervention	Rehabilitation protocol	Definition	Classes	Value	Timepoint	Statistical analysis method
			concomitant superior capsular reconstruction, augmentation procedures or less than 1 year follow-up							
**Galasso, O.**[23]	2024	Retrospective	Patients with degenerative and traumatic injuries, primary and elective isolated type III (a full thickness tear in the upper two-thirds of the tendon) or IV (a complete tear without tendon retraction) subscapularis tear, minimum of 2 months follow-up, between Octobre 2005 and November 2020; Exclusion of patients with revision surgery or prior shoulder surgery, fracture or dislocation of the shoulder, glenohumeral osteoarthritis, neurological disorders of the upper extremities, significant cognitive impairment or failure to understand or complete the questionnaires	One surgeon involved, arthroscopic repair with single-row technique and lasso-loop stitch	Post-operative immobilization in a shoulder abduction brace for 3 weeks, active and passive range of motion exercises of the shoulder started of the immobilization, light strengthening exercises at week 8, sport after 6 months	Image based evaluation with ultrasonography at a minimum of 2-year follow-up, evaluated by two musculoskeletal radiologists using the Sugaya et al, classification adapted for US assessment	Defect (predicted): types IV and V US modified Sugaya et al. Classification - Intact: types I to III	5/45	24	Multivariate linear regression using variables with P values of <.10 in the univariate analysis
**Shibayama, Y.**[62]	2021	Retrospective	Patients with all types of degenerative and traumatic tears, had post-operative MRI at least 2 years after surgery, between June 2010 and October 2015; Exclusion of patients with previous shoulder surgery and/or cuff tear arthropathy, incompletely repaired tendon, irreparable rotator cuff tears or isolated tears of supraspinatus	One junior surgeon involved, subacromial bursectomy with acromioplasty performed, double-row suture bridge technique and medial/lateral row anchors	Post-operative immobilization in a brace for 4 to 6 weeks, the day after the surgery pendulum and deltoid isometric exercises for 4 to 6 weeks, passive forward flexion exercises, passive forward flexion exercises after week 4 to 6, rehabilitation program differed according to the tear size (not precisely described)	Image based evaluation with MRI at 2-year follow-up, evaluated by 2 shoulder fellowship-trained orthopaedic surgeons using Sugaya et al. classification	Defect (predicted): types IV and V Sugaya et al. classification - Intact: types I to III	15/102	24	Multivariate logistic regression with statistically significant values in the univariate analysis

Supplementary Figures 2: Forest plot of individual prognostic factor estimates

Note: Prognostic factors for which at least three association measures were reported were considered for meta-analysis. Meta-analyzed correlation coefficients were reported along with 95% confidence intervals (in black) and 95% prediction intervals (in **dashed blue**).

Figure 2a: Association between increasing corticosteroid injection and repair integrity

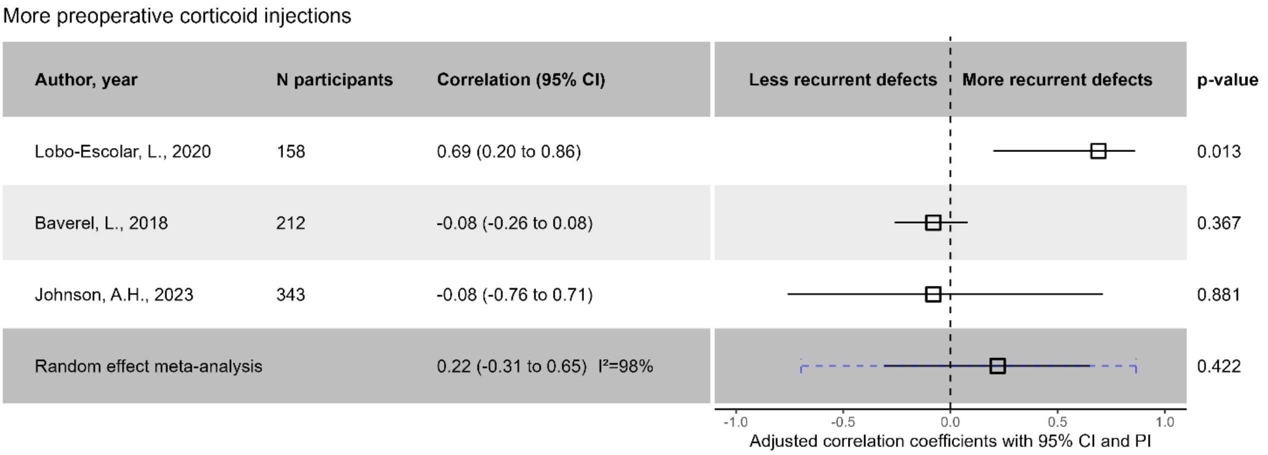

Figure 2b: Association between increased age and repair integrity

Increased age

Author, year	N participants	Correlation (95% CI)	p-value
Lim, T.K., 2021	200	0.06 (0.00 to 0.01)	0.172
Olthof, M.G.L., 2023	97	0.06 (0.01 to 0.11)	0.030
Erşen, A., 2023	94	0.05 (0.01 to 0.10)	0.020
Park, J.S., 2015	339	0.03 (0.01 to 0.06)	0.011
Yeom, J.W., 2023	183	0.03 (0.00 to 0.06)	0.028
Baverel, L., 2018	212	0.03 (-0.01 to 0.06)	0.091
Rashid, M.S., 2017	220	0.03 (0.00 to 0.05)	0.040
Diebold, G., 2017	1600	0.02 (0.01 to 0.03)	0.001
Duong, J.K.H., 2021	1962	0.02 (0.01 to 0.04)	0.001
Le, B.T., 2014	1000	0.02 (0.01 to 0.03)	<0.001
Kwon, J., 2019	531	0.02 (0.00 to 0.04)	0.035
Guo, A. A., 2022	1526	0.01 (0.00 to 0.03)	0.050
Tashjian, R.Z., 2016	72	-0.01 (-0.04 to 0.02)	0.594
Lobo-Escolar, L., 2020	158	-0.03 (-0.09 to 0.03)	0.303
Random effect meta-analysis		0.02 (0.00 to 0.04) I²=0%	0.044

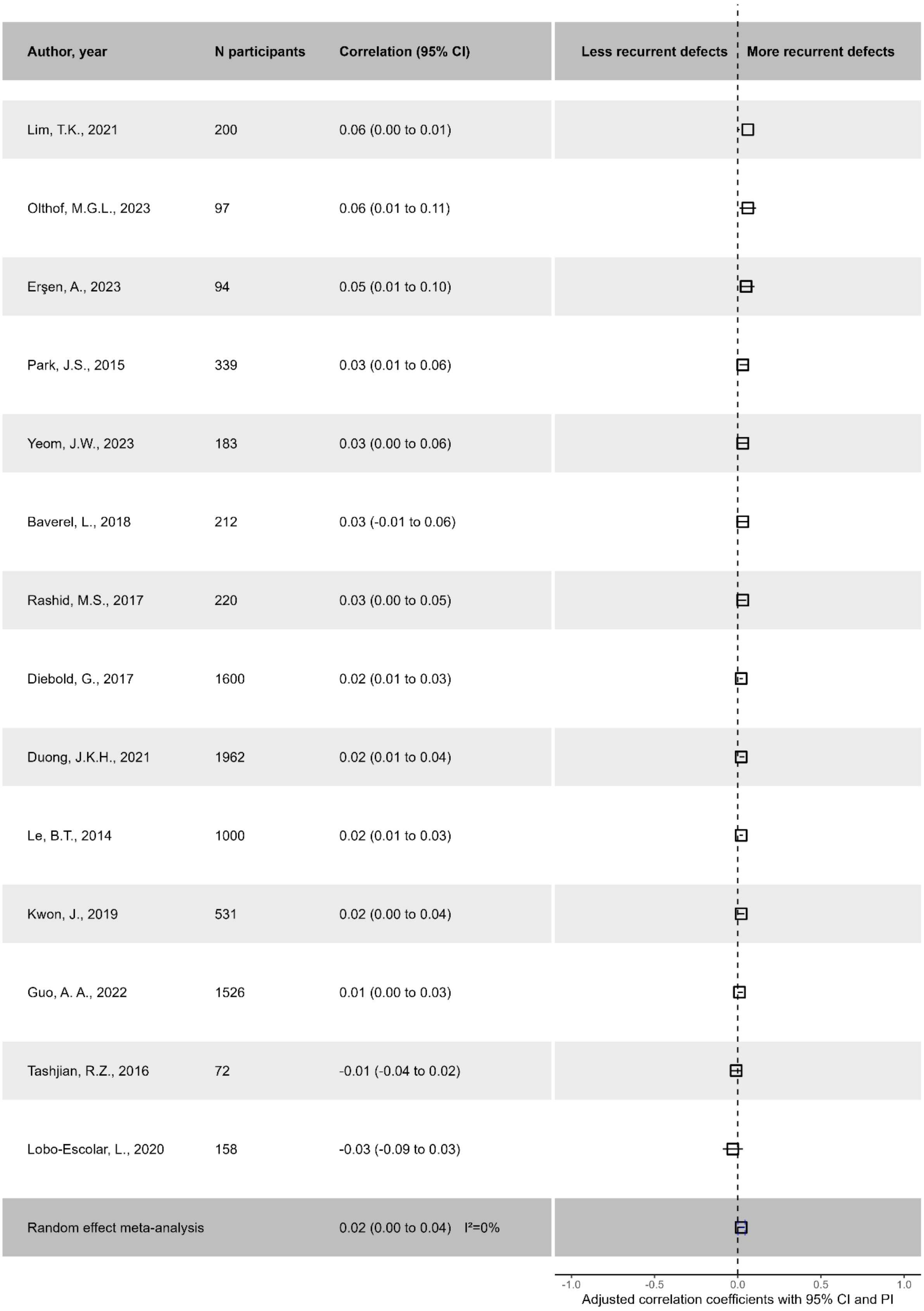

Figure 2c: Association between increasing body mass index and repair integrity

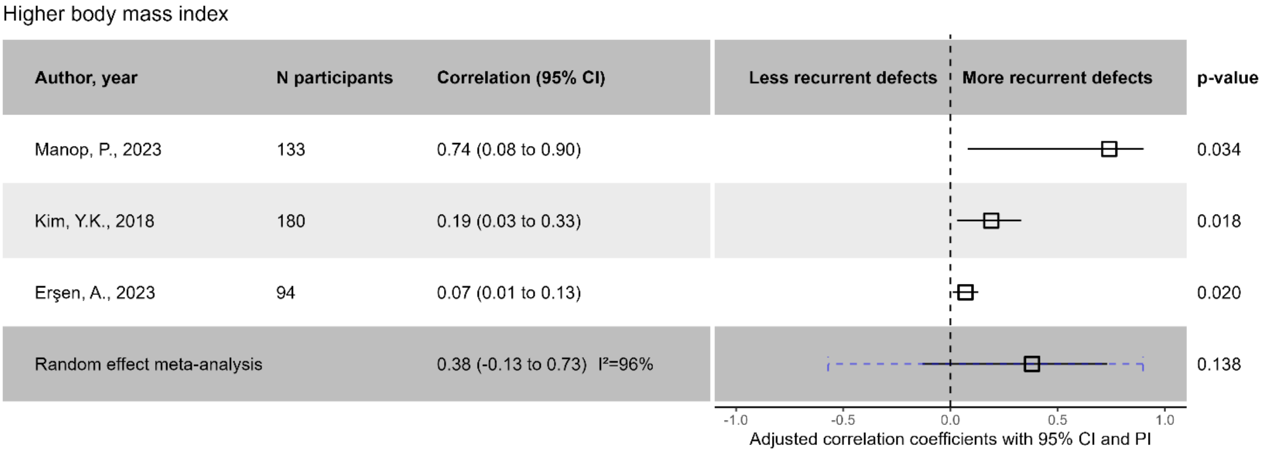

Figure 2d: Association between presence of diabetes and repair integrity

Presence of diabetes

Author, year	N participants	Correlation (95% CI)	p-value
Yeom, J.W., 2023	183	0.65 (0.20 to 0.83)	0.010
Kim, Y.K., 2018	180	0.45 (0.01 to 0.85)	0.046
Kim, M.S., 2023	103	0.25 (-0.50 to 0.74)	0.539
Erşen, A., 2023	94	0.07 (-0.53 to 0.59)	0.900
Lim, T.K., 2021	200	0.06 (-0.02 to 0.12)	0.128
Random effect meta-analysis		0.32 (0.05 to 0.55) I²=93%	0.021

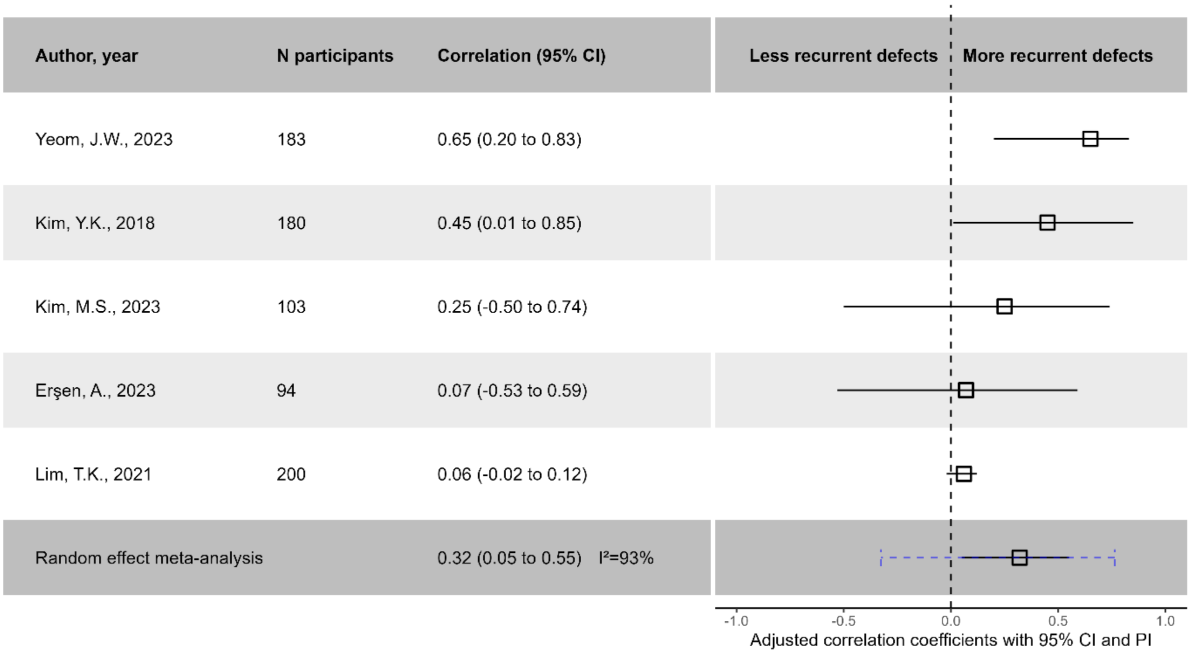

Figure 2e: Association between female sex and repair integrity

Female sex

Author, year	N participants	Correlation (95% CI)	p-value
Lobo-Escolar, L., 2020	158	0.48 (-0.34 to 0.82)	0.240
Baverel, L., 2018	212	0.05 (-0.43 to 0.50)	0.851
Lim, T.K., 2021	200	-0.08 (-0.11 to 0.00)	0.052
Tashjian, R.Z., 2016	72	-0.14 (-0.68 to 0.55)	0.725
Diebold, G., 2017	1600	-0.25 (-0.40 to -0.08)	0.005
Random effect meta-analysis		0.02 (-0.25 to 0.28) I²=96%	0.904

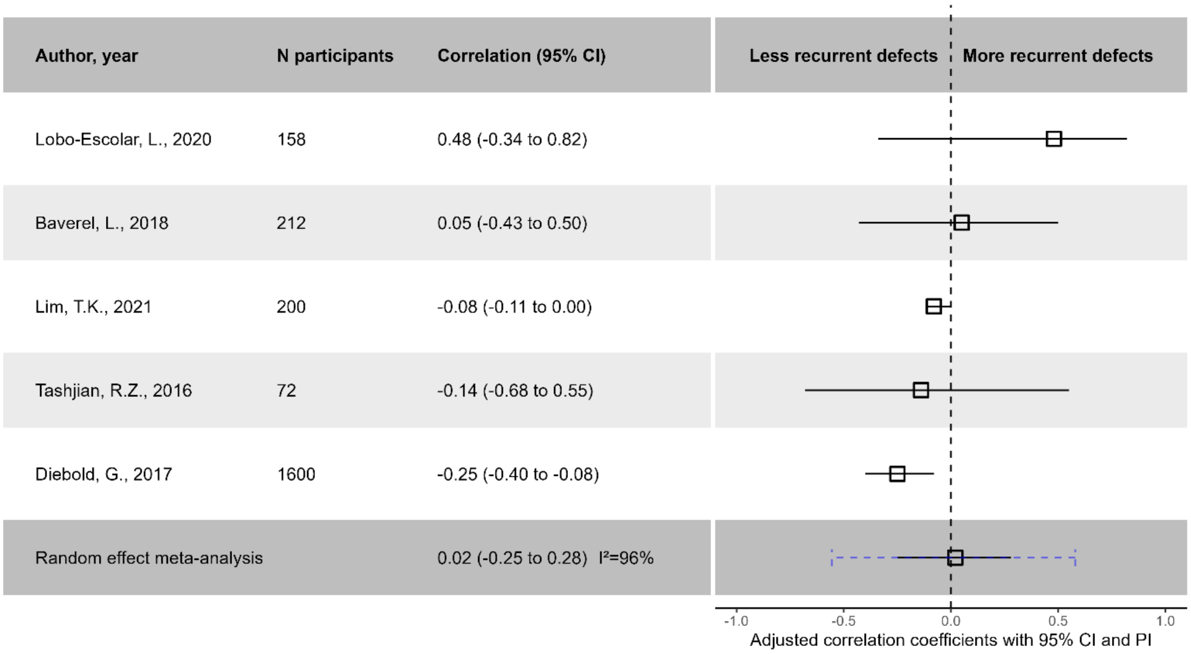

Figure 2f: Association between increasing acromiohumeral distance and repair integrity

Reduced acromiohumeral distance

Author, year	N participants	Correlation (95% CI)	p-value
Park, S.G., 2019	50	0.67 (0.06 to 0.87)	0.013
Liu, X.N., 2018	27	0.37 (0.06 to 0.59)	0.022
Kim, Y.K., 2018	180	0.31 (0.09 to 0.48)	0.006
Lobo-Escolar, L., 2020	158	0.20 (-0.53 to 0.71)	0.634
Caffard, T., 2023	55	0.02 (0.00 to 0.05)	0.010
Random effect meta-analysis		0.32 (0.12 to 0.50) I²=78%	0.002

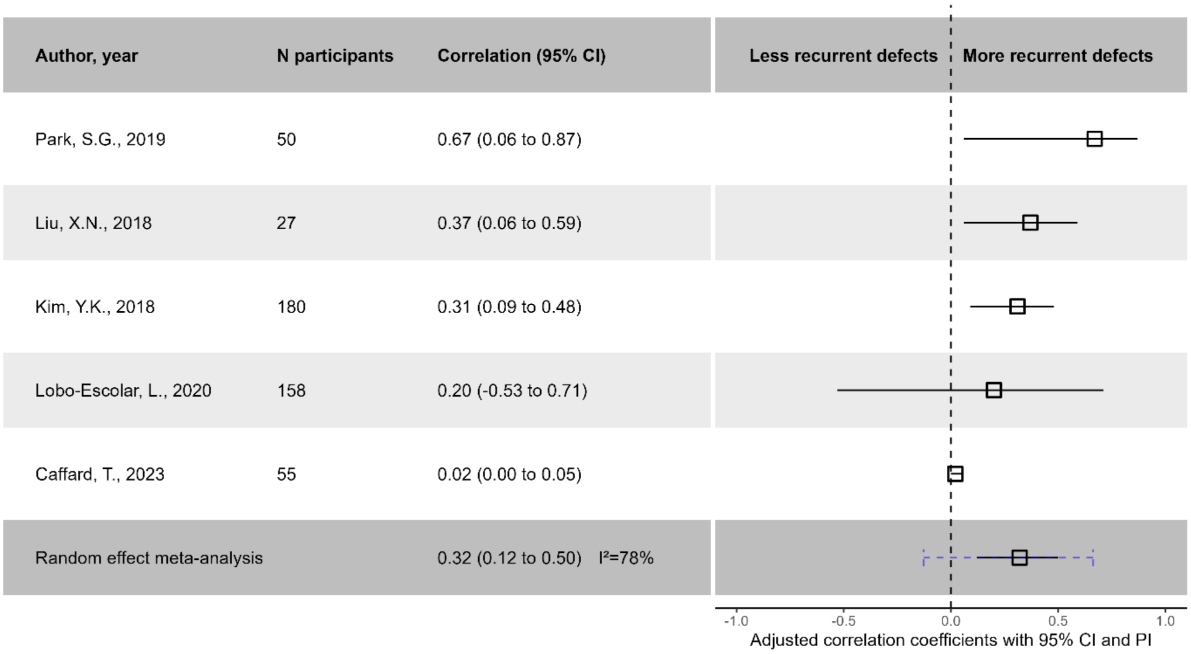

Figure 2g: Association between increasing fatty infiltration of the infraspinatus and repair integrity

Greater fatty infiltration of ISP

Author, year	N participants	Correlation (95% CI)	p-value
Kim, Y.K., 2018	180	0.87 (0.64 to 0.94)	0.005
Park, S.G., 2019	50	0.74 (0.03 to 0.91)	0.063
Park, J.S., 2015	339	0.59 (0.32 to 0.74)	0.001
Kwon, J., 2019	531	0.31 (-0.42 to 0.74)	0.015
Shibayama, Y., 2021	102	0.04 (-0.59 to 0.63)	0.900
Lim, T.K., 2021	200	-0.06 (-0.08 to 0.02)	0.267
Random effect meta-analysis		0.49 (0.15 to 0.73) I²=98%	0.007

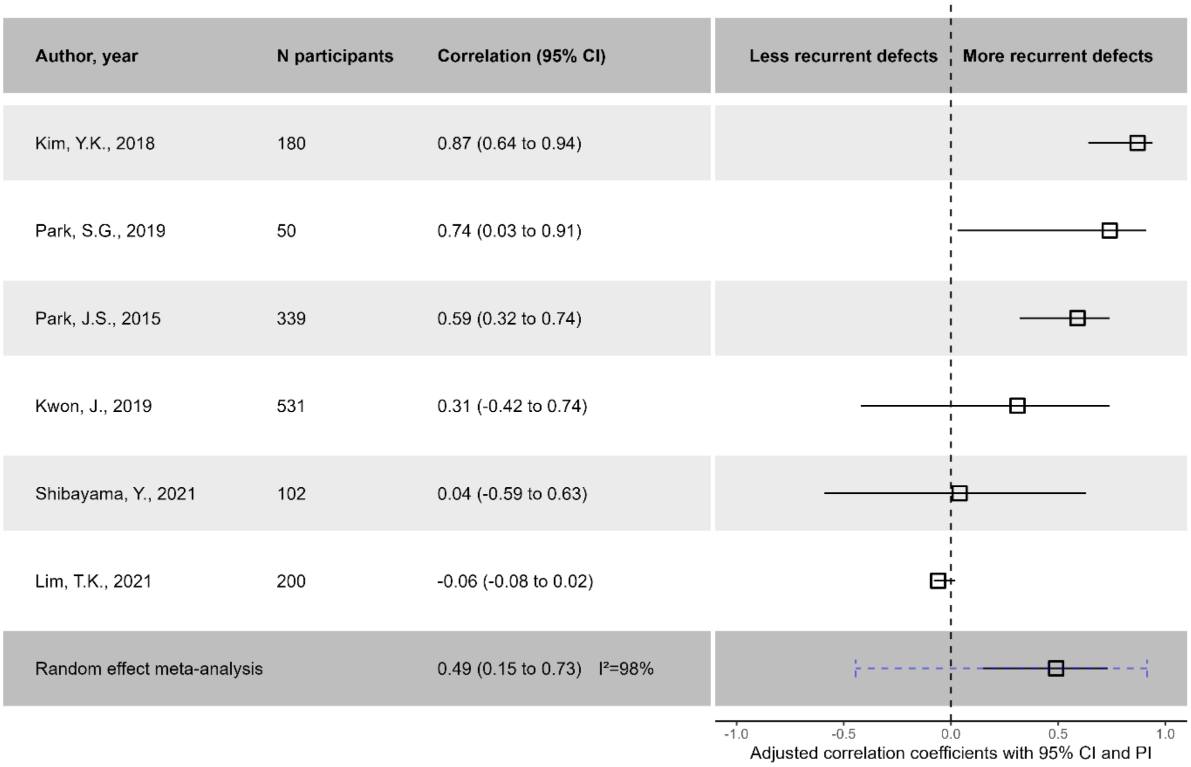

Figure 2h: Association between increasing fatty infiltration of the supraspinatus and repair integrity

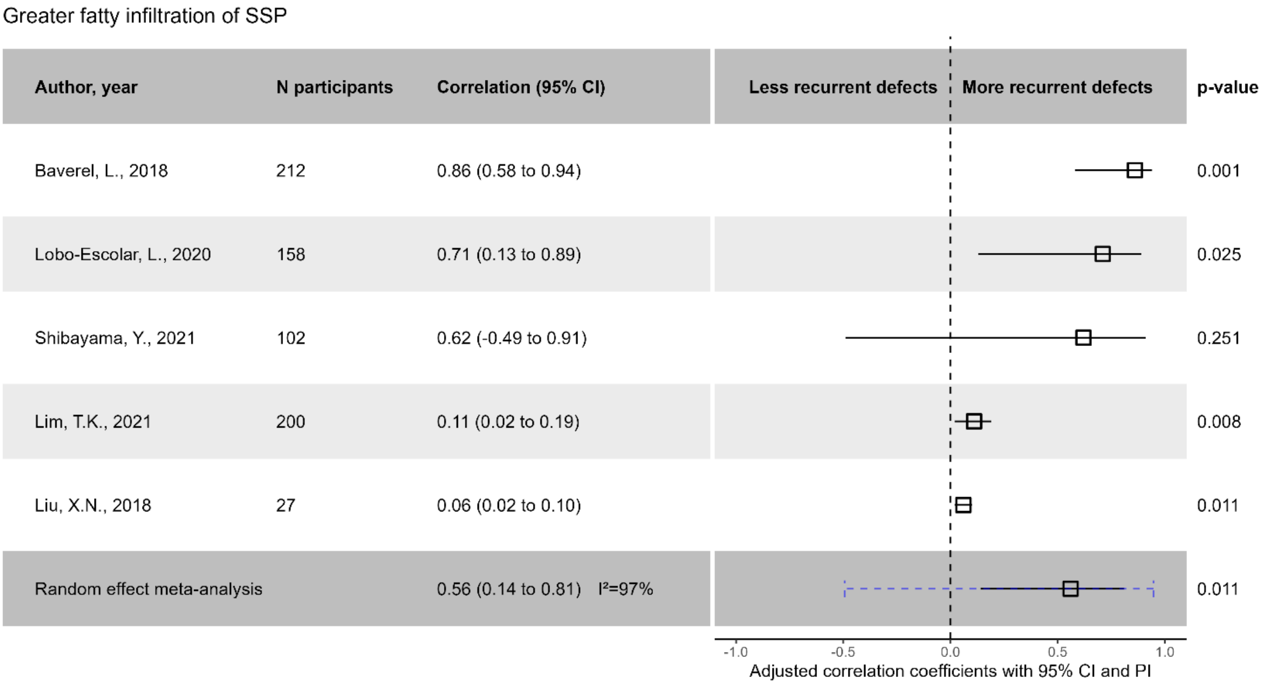

Figure 2i: Association between increasing fatty infiltration and repair integrity

Greater fatty infiltration

Author, year	N participants	Correlation (95% CI)	p-value
Johnson, A.H., 2023	343	0.71 (0.07 to 0.89)	0.034
Choi, S., 2014	147	0.58 (0.34 to 0.72)	0.001
Yeom, J.W., 2023	183	0.30 (0.02 to 0.52)	0.035
Random effect meta-analysis		0.55 (0.26 to 0.75) I²=95%	<0.001

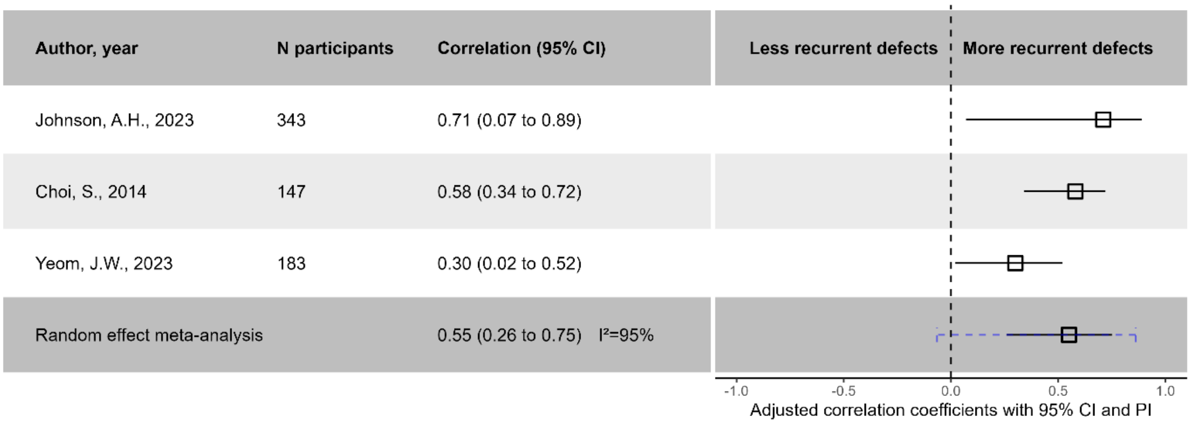

Figure 2j: Association between increasing glenoidal distance and repair integrity

Greater glenoid distance

Author, year	N participants	Correlation (95% CI)	p-value
Kim, Y.K., 2018	180	0.11 (0.00 to 0.22)	0.049
Caffard, T., 2023.1	55	0.00 (-0.11 to 0.17)	0.650
Caffard, T., 2023	55	-0.25 (-0.42 to 0.05)	0.080
Random effect meta-analysis		-0.02 (-0.24 to 0.19) I²=63%	0.821

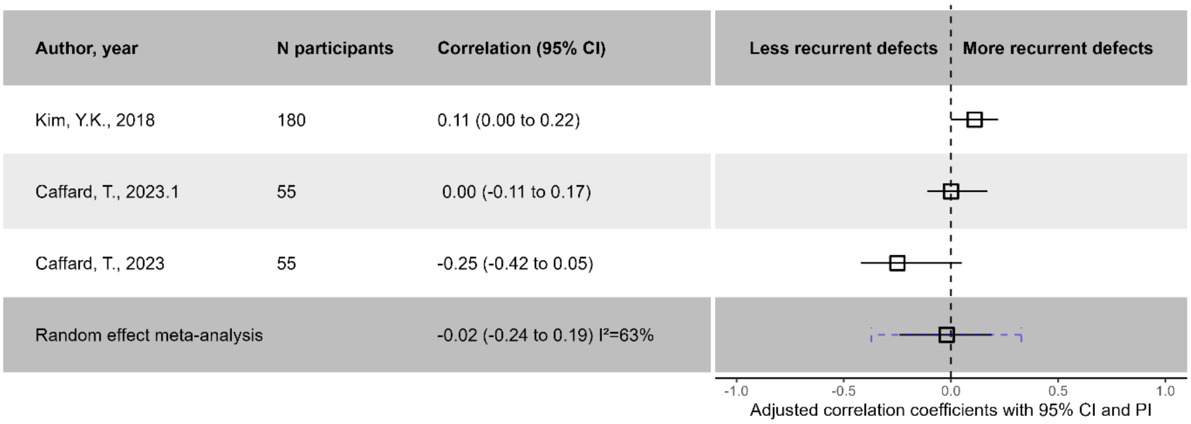

Figure 2k: Association between increasing muscle atrophy of the supraspinatus and repair integrity

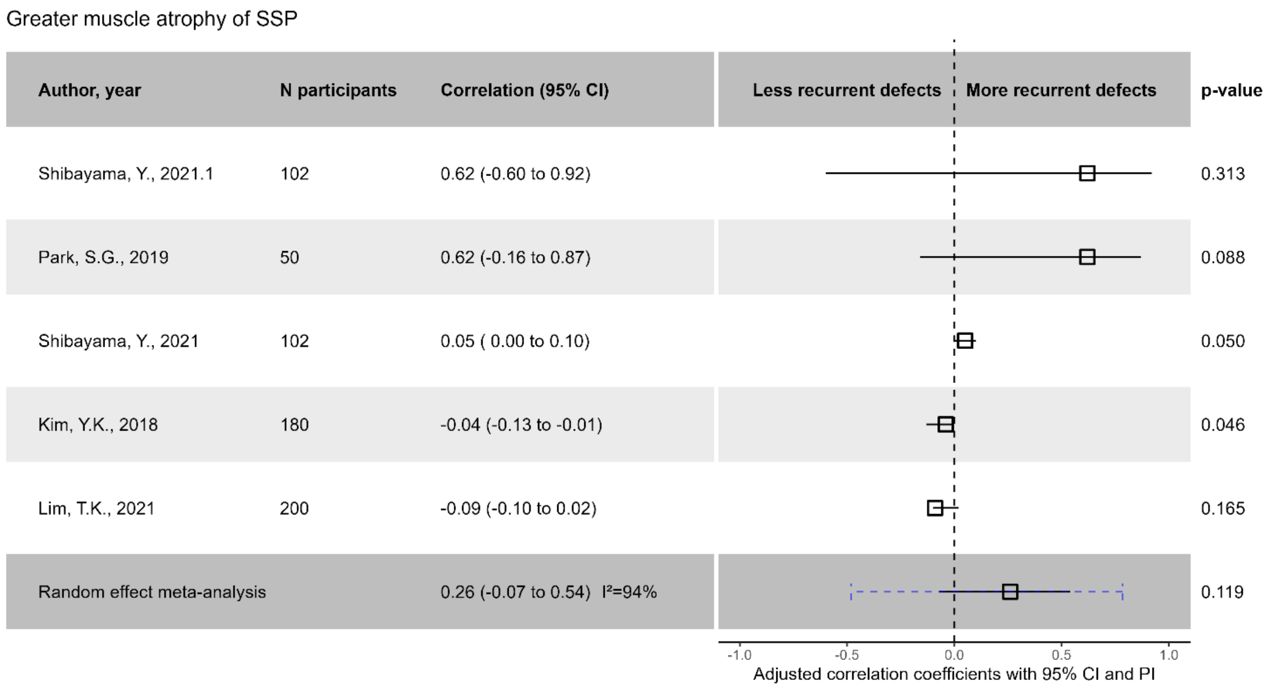

Figure 2l: Association between increasing tear retraction and repair integrity

Greater tear retraction

Author, year	N participants	Correlation (95% CI)	p-value
Kang, Y., 2017	50	0.49 (0.14 to 0.70)	0.008
Park, S.G., 2019	50	0.48 (-0.26 to 0.81)	0.083
Manop, P., 2023	133	0.42 (-0.65 to 0.87)	0.488
Tashjian, R.Z., 2016	72	0.41 (0.06 to 0.64)	0.021
Baverel, L., 2018	212	0.25 (-0.32 to 0.65)	0.388
Kim, Y.K., 2018	180	0.18 (0.05 to 0.29)	0.001
Liu, X.N., 2018	27	0.09 (0.01 to 0.18)	0.034
Kwon, J., 2019	531	0.03 (0.01 to 0.05)	0.001
Random effect meta-analysis		0.29 (0.15 to 0.42) I^2=82%	<0.001

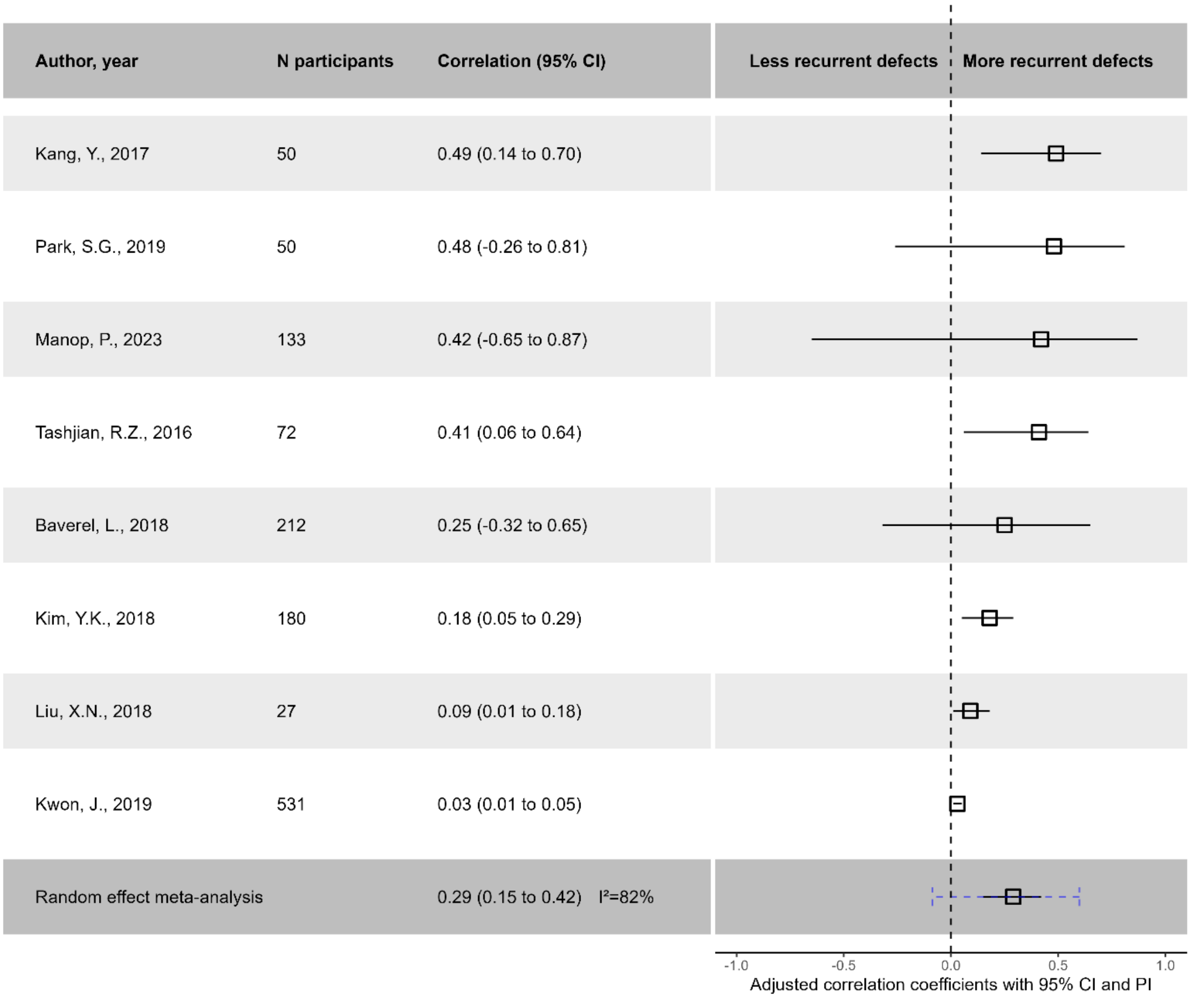

Figure 2m: Association between higher degree of tear severity and repair integrity

Greater tear severity

Author, year	N participants	Correlation (95% CI)	p-value
Lobo-Escolar, L., 2020	158	0.71 (0.11 to 0.89)	0.028
Herring, M.J., 2019	500	0.66 (-0.92 to 1.00)	0.900
Diebold, G., 2017	1600	0.51 (0.33 to 0.64)	0.001
Johnson, A.H., 2023	343	0.43 (-0.04 to 0.70)	0.068
Guo, A. A., 2022	1526	0.09 (0.00 to 0.17)	0.050
Le, B.T., 2014	1000	0.01 (0.00 to 0.03)	0.049
Rashid, M.S., 2017	220	-0.65 (-0.83 to -0.24)	0.010
Random effect meta-analysis		0.28 (-0.01 to 0.53) I²=99%	0.062

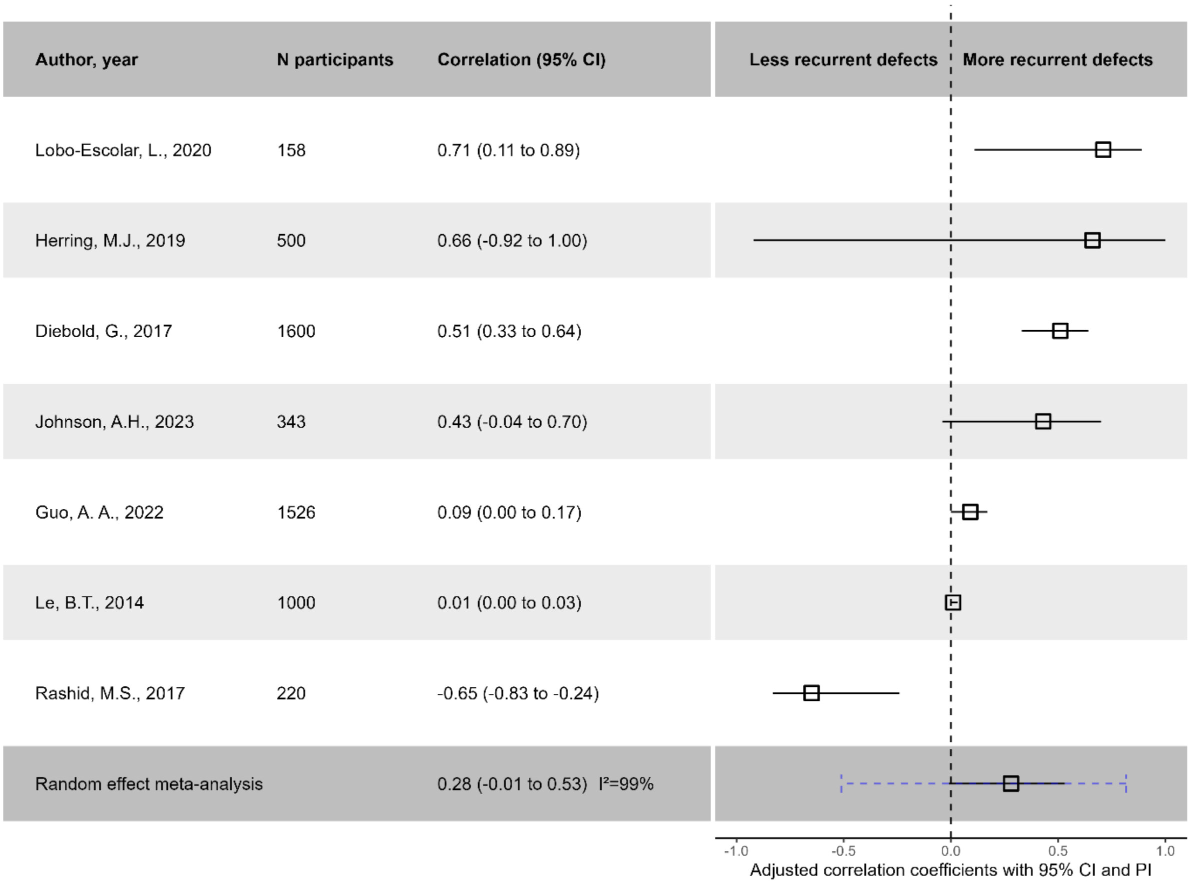

Figure 2n: Association between larger tear size and repair integrity

Larger tear size

Author, year	N participants	Correlation (95% CI)	p-value
Manop, P., 2023	133	0.83 (0.54 to 0.92)	0.001
Chen, Y., 2020	145	0.63 (0.24 to 0.80)	0.005
Shibayama, Y., 2021.2	102	0.62 (0.23 to 0.80)	0.005
Park, J.S., 2015	339	0.47 (0.09 to 0.70)	0.018
Le, B.T., 2014.2	1000	0.45 (0.00 to 0.71)	0.049
Park, S.G., 2019	50	0.43 (-0.39 to 0.81)	0.109
Tokunaga, T., 2023	187	0.40 (0.13 to 0.60)	0.004
Le, B.T., 2014.1	1000	0.36 (0.00 to 0.61)	0.049
Duong, J.K.H., 2021	1962	0.33 (0.00 to 0.57)	0.050
Choi, S., 2014	147	0.31 (-0.01 to 0.55)	0.058
Lim, T.K., 2021.1	200	0.12 (0.00 to 0.01)	0.064
Lim, T.K., 2021	200	0.04 (0.00 to 0.01)	0.496
Shibayama, Y., 2021.1	102	0.01 (-0.13 to 0.16)	0.846
Diebold, G., 2017	1600	0.00 (0.00 to 0.00)	0.001
Shibayama, Y., 2021	102	-0.05 (-0.16 to 0.06)	0.367
Le, B.T., 2014	1000	-0.08 (-0.17 to 0.00)	0.049
Random effect meta-analysis		0.33 (0.21 to 0.45) I²=97%	<0.001

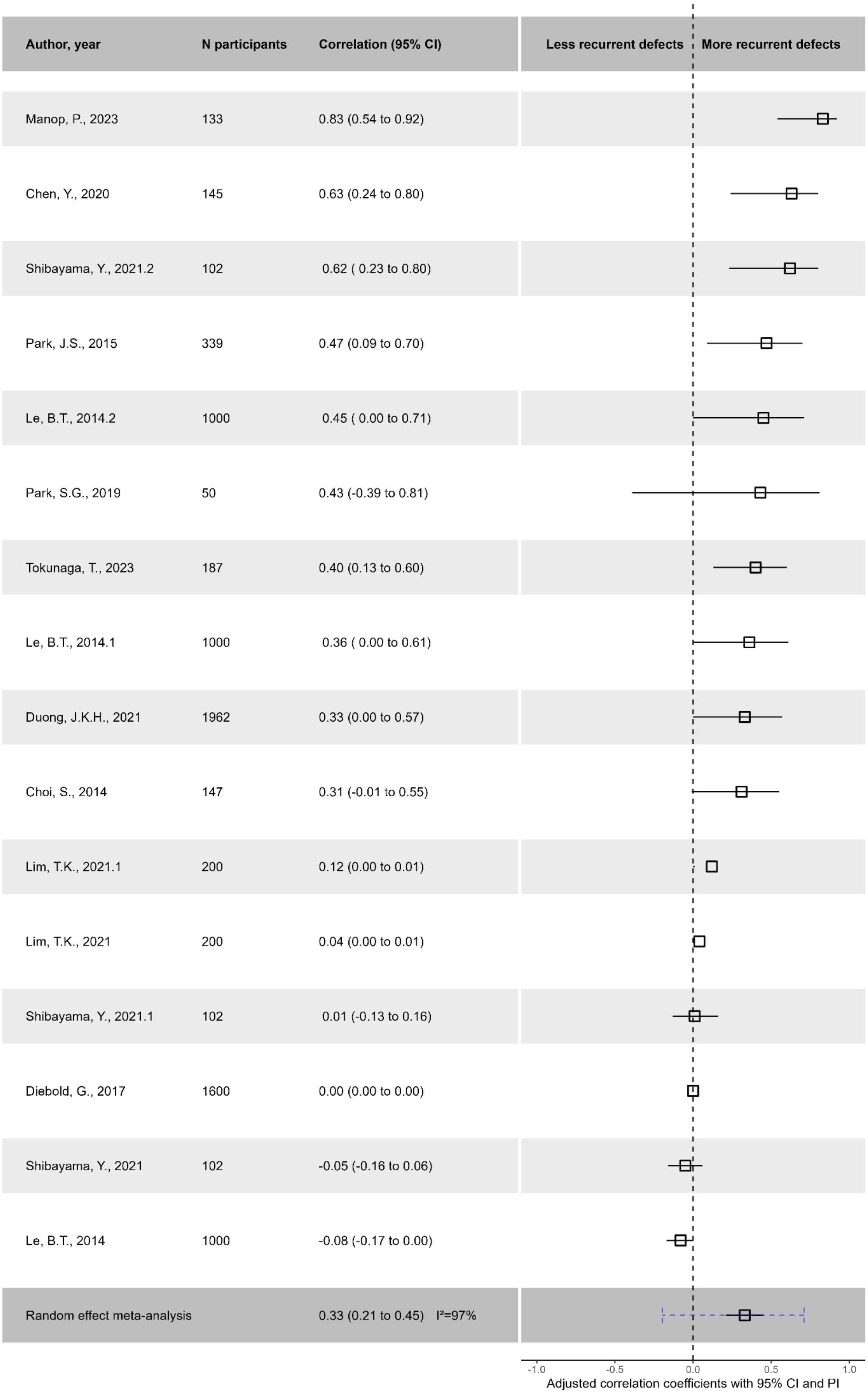

Figure 2o: Association between higher degree of tendon degeneration and repair integrity

Greater tendon degeneration

Author, year	N participants	Correlation (95% CI)	p-value
Kim, Y.K., 2018	180	0.74 (0.16 to 0.90)	0.022
Baverel, L., 2018	212	0.37 (-0.18 to 0.70)	0.184
Tokunaga, T., 2023	187	0.26 (0.13 to 0.40)	0.001
Random effect meta-analysis		0.49 (0.13 to 0.73) I²=96%	0.010

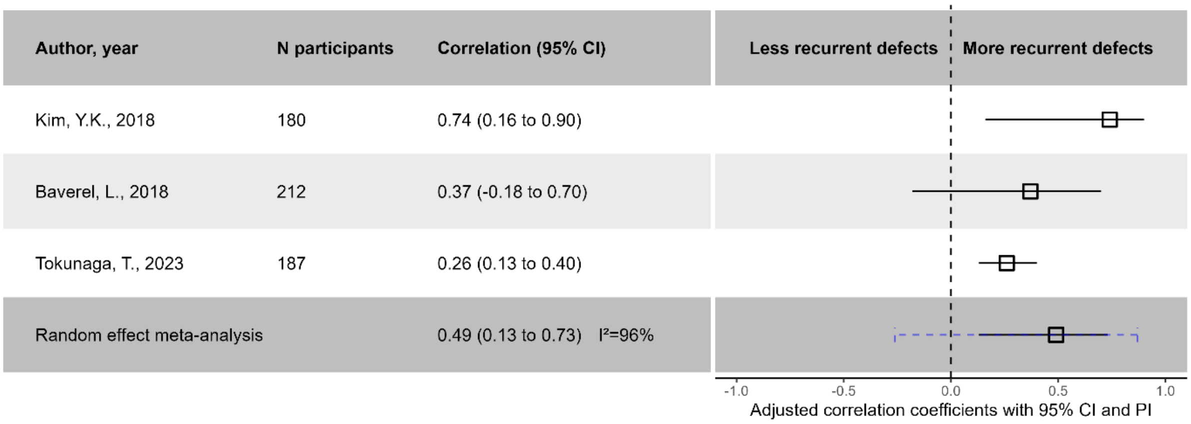

Supplementary Table 3: Risk of bias of included studies according to QUIPS tool

Author	Year	Study participation	Study attrition	Prognostic Factor measurement	Outcome measurement	Study confounding	Statistical analysis and reporting	Overall
**Park, J.S.**[56]	2015	Low	Low	Moderate	Low	Moderate	High	High
**Tan, M.**[69]	2016	Low	Low	Low	Low	Moderate	High	High
**Tashijan, R.Z.**[70]	2016	Moderate	Low	Moderate	Low	Moderate	Moderate	Moderate
**Diebold, G.**[16]	2017	Low	Moderate	Moderate	Moderate	Moderate	Low	Moderate
**Kang, Y.**[35]	2017	High	Moderate	High	High	High	High	High
**Rashid, M.S.**[58]	2017	High	High	High	High	High	High	High
**Baverel, L.**[5]	2018	Low	Low	Low	Moderate	Moderate	Moderate	Moderate
**Kim, Y.K.**[40]	2018	Moderate	Moderate	Moderate	Moderate	Low	Moderate	Moderate
**Liu, X.N.**[45]	2018	Low	Moderate	Low	Low	Moderate	High	High
**Herring, M.J.**[30]	2019	Moderate	Low	High	High	High	High	High
**Kwon, J.**[41]	2019	Moderate	Low	Moderate	Moderate	Moderate	High	High
**McColl, A.H.**[49]	2019	Low	Low	Moderate	High	High	High	High
**Park, S.G.**[57]	2019	Low	Low	Low	High	High	High	High
**Chen, Y.**[8]	2020	Moderate	Low	Low	Moderate	High	High	High
**Choi, S.**[10]	2014	Low	Low	Low	Low	Moderate	High	High
**Le, B.T.**[43]	2014	Low	Low	Moderate	Low	Low	Moderate	Moderate
**Lobo-Escolar, L.**[46]	2020	Low	Low	Moderate	Low	Low	Moderate	Moderate
**Duong, J.K.H.**[17]	2021	Low	Low	Moderate	Moderate	Moderate	Moderate	Moderate
**Lim, T.K.**[44]	2021	Low	Low	Low	Moderate	Moderate	Low	Moderate
**Guo, A.A.**[26]	2022	Moderate	Moderate	High	High	High	High	High
**Caffard, T.**[7]	2023	Low	Low	High	High	High	High	High
**Erşen, A.**[18]	2023	Low	Low	Low	Low	Moderate	Moderate	Moderate
**Johnson, A.H.**[34]	2023	Low	Moderate	High	High	High	High	High
**Kim, M.S.**[39]	2023	Low	Low	Low	Low	Low	Low	Low
**Manop, P.**[48]	2023	Low	High	Low	Moderate	Moderate	Low	High
**Olthof, M.G.L.**[54]	2023	Low	Low	Low	Low	Moderate	Low	Moderate
**Tokunaga, T.**[72]	2023	Low	Low	Low	Low	Low	Low	Low
**Yeom, J.W.**[74]	2023	Low	Low	Low	Low	Moderate	Moderate	Moderate
**Galasso, O.**[23]	2024	Low	Low	Low	Low	High	High	High
**Shibayama, Y.**[62]	2021	Moderate	Moderate	Low	Low	Low	Low	Moderate

Supplementary Table 4: Quality of synthesized evidence regarding prognostic factors estimates

Prognostic factors	Number of		I^2 (%)	Meta-analysed correlation (95% CI)	P value	Study design	Quality of evidence according to GRADE items						Overall quality
							Downgrading items					Upgrading items	
	Participants	Associations					Study limitations	Inconsistency	Indirectness	Imprecision	Publication bias	Moderate/ large effect size	
**Age**	8194	14	0	0.02 (0.00 to 0.04)	0.044	✓	×	✓	✓	✓	✓	✓	●●●●
**Acromiohumeral distance**	470	5	77	0.32 (0.13 to 0.50)	0.002	✓	×	×	✓	✓	×	✓	●●●○
**Fatty infiltration SSP**	699	5	97	0.63 (0.14 to 0.81)	0.011	✓	×	×	✓	✓	×	✓	●●●○
**Tear retraction**	1255	8	82	0.30 (0.15 to 0.42)	<0.001	✓	×	×	✓	✓	×	✓	●●●○
**Tear size**	8269	16	97	0.35 (0.21 to 0.45)	<0.001	✓	×	×	✓	✓	✓	✓	●●●○
**Body mass index**	407	3	96	0.40 (-0.13 to 0.73)	0.138	✓	×	×	✓	✓	×	×	●●○○
**Diabetes**	760	5	93	0.33 (0.05 to 0.55)	0.021	✓	×	×	×	×	×	✓	●●○○
**Fatty infiltration**	673	3	95	0.62 (0.26 to 0.75)	<0.001	✓	×	×	×	✓	×	✓	●●○○
**Fatty infiltration ISP**	1402	6	98	0.54 (0.15 to 0.73)	0.007	✓	×	×	✓	×	×	✓	●●○○
**Female sex**	2242	5	96	0.02 (-0.25 to 0.28)	0.904	✓	×	×	✓	×	×	×	●●○○
**Glenoid distance**	290	3	63	-0.02 (-0.24 to 0.19)	0.821	✓	×	×	✓	✓	×	×	●●○○
**Muscle atrophy SSP**	634	5	94	0.26 (-0.07 to 0.54)	0.119	✓	×	×	×	✓	×	×	●●○○
**Tendon degeneration**	579	3	96	0.53 (0.13 to 0.73)	0.010	✓	×	×	×	×	×	✓	●●○○
**Preoperative corticoid injections**	713	3	98	0.22 (-0.31 to 0.65)	0.422	✓	×	×	×	×	×	×	●○○○
**Tear severity**	5347	7	99	0.29 (-0.01 to 0.53)	0.062	✓	×	×	×	×	×	×	●○○○

Footnote: For GRADE factors: ✓, no serious limitations; ×, moderate or serious limitations (or not present for moderate/large effect size, dose effect); unclear, unable to rate item based on available information**.** Prognostic factors were ranked according to the overall quality of the synthesized evidence: ●○○○: Very low; ●●○○: Low; ●●●○: Moderate; ●●●● High. GRADE factors were used to downgrade or upgrade the certainty of the synthesized evidence. The items study design, study limitations, inconsistency, indirectness, imprecision and publication bias were used to downgrade the certainty. The items moderate/large effect size and plausible confounding were used to upgrade the certainty.

BIBLIOGRAPHY

1. Abtahi AM, Granger EK, Tashjian RZ. Factors affecting healing after arthroscopic rotator cuff repair. World J Orthop 2015;6:211-20. doi:10.5312/wjo.v6.i2.211
2. Audigé L, Aghlmandi S, Grobet C, Stojanov T, Müller AM, Felsch Q, et al. Prediction of shoulder stiffness after arthroscopic rotator cuff repair. The American Journal of Sports Medicine 2021;49:3030-9. doi:https://doi.org/10.1177/03635465211028980
3. Baker WL, Michael White C, Cappelleri JC, Kluger J, Coleman CI, From the Health Outcomes P, et al. Understanding heterogeneity in meta-analysis: the role of meta-regression. International Journal of Clinical Practice 2009;63:1426-34. doi:https://doi.org/10.1111/j.1742-1241.2009.02168.x
4. Barth J, Fotiadis E, Barthelemy R, Genna S, Saffarini M. Ultrasonic evaluation of the repair integrity can predict functional outcomes after arthroscopic double-row rotator cuff repair. Knee Surgery, Sports Traumatology, Arthroscopy 2015;23:376-85. doi:10.1007/s00167-015-3505-z
5. Baverel L, Boutsiadis A, Reynolds RJ, Saffarini M, Barthélémy R, Barth J. Do corticosteroid injections compromise rotator cuff tendon healing after arthroscopic repair? JSES Open Access 2018;2:54-9. doi:https://doi.org/10.1016/j.jses.2017.11.005
6. Bramer WM, Giustini D, de Jonge GB, Holland L, Bekhuis T. De-duplication of database search results for systematic reviews in EndNote. Journal of the Medical Library Association: JMLA 2016;104:240.
7. Caffard T, Kralewski D, Ludwig M, Dornacher D, Fuchs M, Kappe T, et al. High Acromial Slope and Low Acromiohumeral Distance Increase the Risk of Retear of the Supraspinatus Tendon After Repair. Clinical Orthopaedics and Related Research 2023;481:1158-70. doi:10.1097/CORR.0000000000002520
8. Chen Y, Jiang F, Li H, Chen S, Qiao Y, Li Y, et al. Retears and Concomitant Functional Impairments After Rotator Cuff Repair: Shoulder Activity as a Risk Factor. The American Journal of Sports Medicine 2020;48:931-8. doi:10.1177/0363546519900897
9. Cho NS, Moon SC, Jeon JW, Rhee YG. The Influence of Diabetes Mellitus on Clinical and Structural Outcomes After Arthroscopic Rotator Cuff Repair. The American Journal of Sports Medicine 2015;43:991-7. doi:10.1177/0363546514565097
10. Choi S, Kim MK, Kim GM, Roh Y-H, Hwang IK, Kang H. Factors associated with clinical and structural outcomes after arthroscopic rotator cuff repair with a suture bridge technique in medium,

large, and massive tears. Journal of Shoulder and Elbow Surgery 2014;23:1675-81. doi:https://doi.org/10.1016/j.jse.2014.02.021

11. Cohen J, Cohen P, West SG, Aiken LS. Applied multiple regression/correlation analysis for the behavioral sciences. Routledge; 2013. (ISBN No. 0203774442)

12. Collins GS, Moons KGM, Dhiman P, Riley RD, Beam AL, Calster BV, et al. TRIPOD+AI statement: updated guidance for reporting clinical prediction models that use regression or machine learning methods. BMJ 2024;385:e078378. doi:10.1136/bmj-2023-078378

13. Denard PJ, Burkhart SS. The evolution of suture anchors in arthroscopic rotator cuff repair. Arthroscopy: The Journal of Arthroscopic & Related Surgery 2013;29:1589-95.

14. DerSimonian R, Laird N. Meta-analysis in clinical trials. Controlled Clinical Trials 1986;7:177-88. doi:https://doi.org/10.1016/0197-2456(86)90046-2

15. Desmoineaux P. Failed rotator cuff repair. Orthopaedics & Traumatology: Surgery & Research 2019;105:S63-S73. doi:https://doi.org/10.1016/j.otsr.2018.06.012

16. Diebold G, Lam P, Walton J, Murrell GAC. Relationship Between Age and Rotator Cuff Retear: A Study of 1,600 Consecutive Rotator Cuff Repairs. JBJS 2017;99:1198-205. doi:10.2106/jbjs.16.00770

17. Duong JKH, Lam PH, Murrell GAC. Anteroposterior tear size, age, hospital, and case number are important predictors of repair integrity: an analysis of 1962 consecutive arthroscopic single-row rotator cuff repairs. Journal of Shoulder and Elbow Surgery 2021;30:1907-14. doi:https://doi.org/10.1016/j.jse.2020.09.038

18. Erşen A, Şahin K, Albayrak MO. Older age and higher body mass index are independent risk factors for tendon healing in small- to medium-sized rotator cuff tears. Knee surgery, sports traumatology, arthroscopy : official journal of the ESSKA 2023;31:681-90. doi:10.1007/s00167-022-07234-6

19. Fancher AJ, Mok AC, Vopat ML, Templeton K, Kimbrel BK, Tarakemeh A, et al. Comparing Sex-Specific Outcomes After Rotator Cuff Repair: A Meta-analysis. Orthopaedic Journal of Sports Medicine 2022;10:23259671221086259. doi:10.1177/23259671221086259

20. Fermont AJ, Wolterbeek N, Wessel RN, Baeyens JP, de Bie RA. Prognostic factors for successful recovery after arthroscopic rotator cuff repair: a systematic literature review. J Orthop Sports Phys Ther 2014;44:153-63. doi:10.2519/jospt.2014.4832

21. Fisher RA. On the" probable error" of a coefficient of correlation deduced from a small sample. Metron 1921;1:3-32.

22. Foroutan F, Guyatt G, Zuk V, Vandvik PO, Alba AC, Mustafa R, et al. GRADE Guidelines 28: Use of GRADE for the assessment of evidence about prognostic factors: rating certainty in identification of groups of patients with different absolute risks. Journal of Clinical Epidemiology 2020;121:62-70. doi:https://doi.org/10.1016/j.jclinepi.2019.12.023

23. Galasso O, Mercurio M, Gasparini G, Cosentino O, Massarini A, Orlando N, et al. Arthroscopic rotator cuff repair in patients over 65 years of age: successful functional outcomes and a high tendon integrity rate can be obtained after surgery. JSES International 2024;8:299-303. doi:10.1016/j.jseint.2023.11.010

24. Goutallier D, Le Guilloux P, Postel JM, Radier C, Bernageau J, Zilber S. Acromio humeral distance less than six millimeter: Its meaning in full-thickness rotator cuff tear. Orthopaedics & Traumatology: Surgery & Research 2011;97:246-51. doi:https://doi.org/10.1016/j.otsr.2011.01.010

25. Goutallier D, Postel J-M, Gleyze P, Leguilloux P, Van Driessche S. Influence of cuff muscle fatty degeneration on anatomic and functional outcomes after simple suture of full-thickness tears. Journal of Shoulder and Elbow Surgery 2003;12:550-4. doi:https://doi.org/10.1016/S1058-2746(03)00211-8

26. Guo AA, Stitz DJ, Lam P, Murrell GAC. Tear Size and Stiffness Are Important Predictors of Retear: An Assessment of Factors Associated with Repair Integrity at 6 Months in 1,526 Rotator Cuff Repairs. JBJS Open Access 2022;7. doi:10.2106/JBJS.OA.22.00006

27. Haddaway NR, Grainger MJ, Gray CT. Citationchaser: A tool for transparent and efficient forward and backward citation chasing in systematic searching. Research Synthesis Methods 2022;13:533-45. doi:https://doi.org/10.1002/jrsm.1563

28. Harris PA, Taylor R, Minor BL, Elliott V, Fernandez M, O'Neal L, et al. The REDCap consortium: Building an international community of software platform partners. Journal of biomedical informatics 2019;95:103208. doi:https://doi.org/10.1016/j.jbi.2019.103208

29. Hayden JA, Côté P, Bombardier C. Evaluation of the quality of prognosis studies in systematic reviews. Annals of internal medicine 2006;144:427-37.

30. Herring MJ, White M, Braman JP. The WORC Index and Predicting Treatment Failure in Patients Undergoing Primary Arthroscopic Rotator Cuff Repair. Orthopaedic Journal of Sports Medicine 2019;7:2325967119859518. doi:10.1177/2325967119859518

31. Hirt J, Nordhausen T, Fuerst T, Ewald H, Appenzeller-Herzog C. Guidance on terminology, application, and reporting of citation searching: the TARCiS statement. bmj 2024;385.

32. Huguet A, Hayden JA, Stinson J, McGrath PJ, Chambers CT, Tougas ME, et al. Judging the quality of evidence in reviews of prognostic factor research: adapting the GRADE framework. Systematic reviews 2013;2:1-12.

33. Jayakumar P, Bozic KJ. Advanced decision-making using patient-reported outcome measures in total joint replacement. Journal of Orthopaedic Research 2020;38:1414-22. doi:https://doi.org/10.1002/jor.24614

34. Johnson AH, West M, Fowler MB, Petre BM, Turcotte JJ, Redziniak DE. What is the Optimal Construct to Reduce Failure in Arthroscopic Four Anchor Rotator Cuff Repair? Shoulder And Elbow 2023;15:33-9. doi:10.1177/17585732221076066

35. Kang Y, Lee GY, Lee JW, Lee E, Kim B, Kim SJ, et al. Texture Analysis of Torn Rotator Cuff on Preoperative Magnetic Resonance Arthrography as a Predictor of Postoperative Tendon Status. Korean J Radiol 2017;18:691-8. doi:10.3348/kjr.2017.18.4.691

36. Keener JD. Revision Rotator Cuff Repair. Clinics in Sports Medicine 2012;31:713-25. doi:10.1016/j.csm.2012.07.007

37. Kholinne E, Kwak JM, Sun Y, Kim H, Park D, Koh KH, et al. The relationship between rotator cuff integrity and acromiohumeral distance following open and arthroscopic rotator cuff repair. Sicot j 2021;7:23. doi:10.1051/sicotj/2021012

38. Kim DH, Jang YH, Choi YE, Lee H-R, Kim SH. Evaluation of Repair Tension in Arthroscopic Rotator Cuff Repair:Does It Really Matter to the Integrity of the Rotator Cuff? The American Journal of Sports Medicine 2016;44:2807-12. doi:10.1177/0363546516651831

39. Kim MS, Rhee SM, Cho NS. Increased HbA1c Levels in Diabetics During the Postoperative 3-6 Months After Rotator Cuff Repair Correlated With Increased Retear Rates. Arthroscopy - Journal of Arthroscopic and Related Surgery 2023;39:176-82. doi:10.1016/j.arthro.2022.08.021

40. Kim Y-K, Jung K-H, Kim J-W, Kim U-S, Hwang D-H. Factors affecting rotator cuff integrity after arthroscopic repair for medium-sized or larger cuff tears: a retrospective cohort study. Journal of Shoulder and Elbow Surgery 2018;27:1012-20. doi:https://doi.org/10.1016/j.jse.2017.11.016

41. Kwon J, Lee YH, Kim SH, Ko JH, Park BK, Oh JH. Delamination Does Not Affect Outcomes After Arthroscopic Rotator Cuff Repair as Compared With Nondelaminated Rotator Cuff Tears: A Study of 1043 Consecutive Cases. The American Journal of Sports Medicine 2019;47:674-81. doi:10.1177/0363546518817764

42. Lambers Heerspink FO, Dorrestijn O, van Raay JJ, Diercks RL. Specific patient-related prognostic factors for rotator cuff repair: a systematic review. J Shoulder Elbow Surg 2014;23:1073-80. doi:10.1016/j.jse.2014.01.001

43. Le BTN, Wu XL, Lam PH, Murrell GAC. Factors Predicting Rotator Cuff Retears:An Analysis of 1000 Consecutive Rotator Cuff Repairs. The American Journal of Sports Medicine 2014;42:1134-42. doi:10.1177/0363546514525336

44. Lim TK, Bae KH, Choi YS, Kim JH, Yoo JC. Clinical outcome and repair integrity after arthroscopic rotator cuff repair significantly improved during the surgeon's learning curve. Journal of Shoulder and Elbow Surgery 2021;30:1881-90. doi:https://doi.org/10.1016/j.jse.2020.10.031

45. Liu XN, Yang C-J, Lee GW, Kim SH, Yoon Y-H, Noh K-C. Functional and Radiographic Outcomes After Arthroscopic Transosseous Suture Repair of Medium Sized Rotator Cuff Tears. Arthroscopy: The Journal of Arthroscopic & Related Surgery 2018;34:50-7. doi:https://doi.org/10.1016/j.arthro.2017.07.035

46. Lobo-Escolar L, Ramazzini-Castro R, Codina-Grañó D, Lobo E, Minguell-Monyart J, Ardèvol J. Risk factors for symptomatic retears after arthroscopic repair of full-thickness rotator cuff tears. Journal of Shoulder and Elbow Surgery 2021;30:27-33. doi:https://doi.org/10.1016/j.jse.2020.05.010

47. Long Z, Nakagawa K, Wang Z, Amadio PC, Zhao C, Gingery A. Age-related cellular and microstructural changes in the rotator cuff enthesis. Journal of Orthopaedic Research 2022;40:1883-95. doi:https://doi.org/10.1002/jor.25211

48. Manop P, Apivatgaroon A, Puntu W, Chernchujit B. Risk Factors for Rotator Cuff Repair Failure and Reliability of the Rotator Cuff Healing Index (RoHI) in Thai Patients: Comparison of the RoHI With a

Modified Scoring System. Orthopaedic Journal of Sports Medicine 2023;11. doi:10.1177/23259671231179449

49. McColl AH, Lam PH, Murrell GAC. Are we getting any better? A study on repair integrity in 1600 consecutive arthroscopic rotator cuff repairs. JSES Open Access 2019;3:12-20. doi:10.1016/j.jses.2019.01.002

50. McElvany MD, McGoldrick E, Gee AO, Neradilek MB, Matsen FA. Rotator Cuff Repair:Published Evidence on Factors Associated With Repair Integrity and Clinical Outcome. The American Journal of Sports Medicine 2015;43:491-500. doi:10.1177/0363546514529644

51. Monteleone AS, Salerno M, Mondini Trissino da Lodi C, Gonalba GC, Candrian C, Filardo G. The influence of sex is a neglected focus in rotator cuff repair: A systematic review and meta-analysis. Knee Surgery, Sports Traumatology, Arthroscopy 2024;32:2699-710. doi:https://doi.org/10.1002/ksa.12201

52. Moons KG, de Groot JA, Bouwmeester W, Vergouwe Y, Mallett S, Altman DG, et al. Critical appraisal and data extraction for systematic reviews of prediction modelling studies: the CHARMS checklist. PLoS medicine 2014;11:e1001744.

53. Müller AM, Flury M, Alsayed HN, Audigé L. Influence of patient and diagnostic parameters on reported retear rates after arthroscopic rotator cuff repair. Knee surgery, sports traumatology, arthroscopy : official journal of the ESSKA 2017;25:2089-99. doi:10.1007/s00167-017-4481-2

54. Olthof MGL, Flück M, Borbas P, Valdivieso P, Toigo M, Egli F, et al. Structural Musculotendinous Parameters That Predict Failed Tendon Healing After Rotator Cuff Repair. Orthopaedic Journal of Sports Medicine 2023;11. doi:10.1177/23259671231196875

55. Page MJ, McKenzie JE, Bossuyt PM, Boutron I, Hoffmann TC, Mulrow CD, et al. The PRISMA 2020 statement: An updated guideline for reporting systematic reviews. Journal of Clinical Epidemiology 2021;134:178-89. doi:https://doi.org/10.1016/j.jclinepi.2021.03.001

56. Park JS, Park HJ, Kim SH, Oh JH. Prognostic Factors Affecting Rotator Cuff Healing After Arthroscopic Repair in Small to Medium-sized Tears. The American Journal of Sports Medicine 2015;43:2386-92. doi:10.1177/0363546515594449

57. Park S-G, Shim B-J, Seok H-G. How Much Will High Tension Adversely Affect Rotator Cuff Repair Integrity? Arthroscopy: The Journal of Arthroscopic & Related Surgery 2019;35:2992-3000. doi:https://doi.org/10.1016/j.arthro.2019.05.049

58. Rashid MS, Cooper C, Cook J, Cooper D, Dakin SG, Snelling S, et al. Increasing age and tear size reduce rotator cuff repair healing rate at 1 year. Acta Orthopaedica 2017;88:606-11. doi:10.1080/17453674.2017.1370844

59. Razmjou H, Veronica P, Monique C, Susan R, and Kennedy D. Reduced acromiohumeral distance and increased critical shoulder angle: implications for primary care clinicians. The Physician and Sportsmedicine 2020;48:312-9. doi:10.1080/00913847.2019.1703475

60. Riley RD, Moons KG, Snell KI, Ensor J, Hooft L, Altman DG, et al. A guide to systematic review and meta-analysis of prognostic factor studies. bmj 2019;364.

61. Riley RD, van der Windt D, Croft P, Moons KG. Prognosis research in healthcare: concepts, methods, and impact. Oxford University Press; 2019. (ISBN No. 0192516655)

62. Shibayama Y, Hirose T, Sugi A, Mizushima E, Watanabe Y, Tomii R, et al. Relationship between preoperative size of rotator cuff tears measured using radial-slice magnetic resonance images and postoperative rotator cuff integrity: a prospective case-control study. JSES international 2021;6:279-86. doi:10.1016/j.jseint.2021.11.005

63. Steyerberg EW. Clinical Prediction Models. 2009. (ISBN No. 978-0-387-77243-1

978-0-387-77244-8)

64. Stojanov T, Aghlmandi S, Müller AM, Scheibel M, Flury M, Audigé L. Development and internal validation of a model predicting patient-reported shoulder function after arthroscopic rotator cuff repair in a Swiss setting. Diagnostic and Prognostic Research 2023;7:21. doi:10.1186/s41512-023-00156-y

65. Stojanov T, Audigé L, Modler L, Aghlmandi S, Appenzeller-Herzog C, Loucas R, et al. Prognostic factors for improvement of shoulder function after arthroscopic rotator cuff repair: a systematic review. JSES International 2023;7:50-7. doi:10.1016/j.jseint.2022.09.003

66. Stojanov T, Modler L, Müller AM, Aghlmandi S, Appenzeller-Herzog C, Loucas R, et al. Prognostic factors for the occurrence of post-operative shoulder stiffness after arthroscopic rotator cuff repair: a

systematic review. BMC Musculoskeletal Disorders 2022;23:1-10. doi:https://doi.org/10.1186/s12891-022-05030-4

67. Sugaya H, Maeda K, Matsuki K, Moriishi J. Functional and Structural Outcome After Arthroscopic Full-Thickness Rotator Cuff Repair: Single-Row Versus Dual-Row Fixation. Arthroscopy: The Journal of Arthroscopic & Related Surgery 2005;21:1307-16. doi:https://doi.org/10.1016/j.arthro.2005.08.011

68. Sugaya H, Maeda K, Matsuki K, Moriishi J. Repair Integrity and Functional Outcome After Arthroscopic Double-Row Rotator Cuff Repair: A Prospective Outcome Study. JBJS 2007;89:953-60. doi:10.2106/jbjs.F.00512

69. Tan M, Lam PH, Le B, Murrell GAC. Trauma versus no trauma: Effect of tear mechanism in the outcomes of arthroscopic rotator cuff repair in 1300 consecutive patients. Journal of Science and Medicine in Sport 2014;18:e161-NA. doi:10.1016/j.jsams.2014.11.186

70. Tashjian RZ, Granger EK, Zhang Y, Teerlink CC, Cannon-Albright LA. Identification of a genetic variant associated with rotator cuff repair healing. Journal of Shoulder and Elbow Surgery 2016;25:865-72. doi:https://doi.org/10.1016/j.jse.2016.02.019

71. Tashjian RZ, Hung M, Burks RT, Greis PE. Influence of Preoperative Musculotendinous Junction Position on Rotator Cuff Healing Using Single-Row Technique. Arthroscopy: The Journal of Arthroscopic & Related Surgery 2013;29:1748-54. doi:https://doi.org/10.1016/j.arthro.2013.08.014

72. Tokunaga T, Karasugi T, Tanimura S, Miyamoto T. Association of Severe Histological Degeneration of the Torn Supraspinatus Tendon and Retear After Arthroscopic Repair of Full-Thickness Rotator Cuff Tears Using the Suture Bridge Technique. The American journal of sports medicine 2023;51:2411-21. doi:10.1177/03635465231178294

73. Viechtbauer W. Conducting meta-analyses in R with the metafor package. Journal of statistical software 2010;36:1-48.

74. Yeom JW, Kholinne E, Kim DM, Lee JB, Hui B, AlAhmadi BM, et al. Postoperative HbA1c Level as a Predictor of Rotator Cuff Integrity After Arthroscopic Rotator Cuff Repair in Patients With Type 2 Diabetes. Orthopaedic Journal of Sports Medicine 2023;11. doi:10.1177/23259671221145987

75. Zhang AL, Montgomery SR, Ngo SS, Hame SL, Wang JC, Gamradt SC. Analysis of Rotator Cuff Repair Trends in a Large Private Insurance Population. Arthroscopy: The Journal of Arthroscopic & Related Surgery 2013;29:623-9. doi:https://doi.org/10.1016/j.arthro.2012.11.004

76. Zsidai B, Hilkert A-S, Kaarre J, Narup E, Senorski EH, Grassi A, et al. A practical guide to the implementation of AI in orthopaedic research – part 1: opportunities in clinical application and overcoming existing challenges. Journal of Experimental Orthopaedics 2023;10:117. doi:10.1186/s40634-023-00683-z
